# Variation of Antigen 43 self-association modulates bacterial compacting within aggregates and biofilms

**DOI:** 10.1038/s41522-022-00284-1

**Published:** 2022-04-08

**Authors:** Julieanne L. Vo, Gabriela C. Martínez Ortiz, Makrina Totsika, Alvin W. Lo, Steven J. Hancock, Andrew E. Whitten, Lilian Hor, Kate M. Peters, Valentin Ageorges, Nelly Caccia, Mickaël Desvaux, Mark A. Schembri, Jason J. Paxman, Begoña Heras

**Affiliations:** 1 Department of Biochemistry and Chemistry, La Trobe Institute for Molecular Science, La Trobe University, Melbourne, VIC 3086 Australia; 2 Centre for Immunology and Infection Control, School of Biomedical Sciences, Queensland University of Technology, Herston, QLD 4006 Australia; 3 School of Chemistry and Molecular Biosciences, and Australian Infectious Diseases Research Centre, The University of Queensland, Brisbane, QLD 4072 Australia; 4 Australian Centre for Neutron Scattering, Australian Nuclear Science and Technology Organisation, Lucas Heights, NSW 2234 Australia; 5 Université Clermont Auvergne, INRAE, UMR454 MEDiS, 63000 Clermont-Ferrand, France

**Keywords:** Biofilms, Bacteriology

## Abstract

The formation of aggregates and biofilms enhances bacterial colonisation and infection progression by affording protection from antibiotics and host immune factors. Despite these advantages there is a trade-off, whereby bacterial dissemination is reduced. As such, biofilm development needs to be controlled to suit adaptation to different environments. Here we investigate members from one of largest groups of bacterial adhesins, the autotransporters, for their critical role in the assembly of bacterial aggregates and biofilms. We describe the structural and functional characterisation of autotransporter Ag43 variants from different *Escherichia coli* pathotypes. We show that specific interactions between amino acids on the contacting interfaces of adjacent Ag43 proteins drives a common mode of trans-association that leads to cell clumping. Furthermore, subtle variation of these interactions alters aggregation kinetics and the degree of compacting within cell clusters. Together, our structure–function investigation reveals an underlying molecular basis for variations in the density of bacterial communities.

## Introduction

Many bacterial species have the ability to transition from a free-swimming lifestyle to the formation of aggregated or biofilm communities^1^. These multicellular structures offer several adaptation advantages, such as enhanced resistance against antimicrobial agents, chemical detergents and immune factors^2^ and account for 65–80% of bacterial infections in humans^3^. Although very common and often competitively beneficial to bacteria, the molecular mechanisms underlying bacterial self-recognition and aggregation remain to be fully elucidated. For example, there is increasing evidence that bacterial aggregation can be coordinated by controlled chemotactic motility^4–7^ as well as entropic forces that drive the depletion of substances which block cell–cell interactions^8^. In relation to this study, bacterial aggregation is also known to be facilitated by proteins that decorate the cell surface^2^, but the precise motifs and mechanisms of self-recognition remain largely uncharacterised.

A class of proteins often associated with the bacterial aggregation phenotype is the autotransporter (AT) superfamily^2,9^. These proteins belong to the largest group of secreted and outer-membrane proteins in Gram-negative bacteria and share a common translocation pathway defined by the type V secretion system^9–11^. Within this family, the self-associating autotransporters (SAATs), are principally involved in bacterial aggregation and biofilm formation^12^.

The best-characterised SAAT, antigen 43 (Ag43), is a protein that contributes to cell aggregation and biofilm formation in commensal *Escherichia coli* K-12 as well as in major pathotypes including enterohemorrhagic *E. coli* (EHEC) and uropathogenic *E. coli* (UPEC)^9,13–17^. Ag43 possesses a modular domain organisation that is common throughout the ATs, which encompasses an N-terminal signal sequence that directs the secretion of the protein across the inner membrane, a passenger (α) domain that is responsible for the function of the protein, and a C-terminal translocator (β) domain that facilitates the translocation of the functional α-domain to the cell surface^18–21^. After translocation, the Ag43 α-domain (α^43^) is cleaved but remains attached to the cell via non-covalent interactions^9,13,22–25^.

Ag43 exhibits allelic variation based on sequence diversity, particularly within its functional α-domain, and this can lead to altered degrees of aggregation^15,22^. A recent comprehensive analysis of Ag43 functional α-domains identified four major phylogenetic groups (C1–C4) based on amino acid sequence variation, with diverse auto-aggregative properties observed among individual proteins across and within different groups^22^. In the case of the Ag43a variant, which belongs to the C3 group, structural analysis has revealed it possesses an L-shaped β-helical conformation that forms a ‘head-to-tail’ self-association interaction between neighbouring cells to drive bacterial aggregation^13^. However, whether this head-to-tail mechanism of self-association is conserved across all Ag43 variants and other ATs in general, and how sequence changes impact these interactions, remain unanswered questions.

In this study, we have determined the crystal structure of Ag43 variants from three strains representing two diverse *E. coli* pathotypes: the EHEC O157:H7 strain EDL933 and the UPEC strains UTI89 and CFT073. These prototype *E. coli* pathogens cause significant human infections, including haemorrhagic colitis and haemolytic-uraemic syndrome by EHEC^26–28^, and urinary tract and bloodstream infections by UPEC^29^. Importantly, together they encode Ag43 variants that represent different aspects of its phylogenetic diversity. Our data demonstrates that the functional α-domain of these Ag43 proteins folds into a bent three-stranded β-helix, which could be a common feature throughout the SAAT family^18,30,31^. Through structural and functional studies, we have identified the residues on the surface of each Ag43 α-domain that mediate trans-association with other α^43^ molecules, which leads to bacterial clumping. Our results also uncovered subtle differences in α^43^–α^43^ oligomerisation modes, which determine distinct aggregation kinetics and interaction strengths. Overall, we demonstrate a common mode of self-association among Ag43 proteins that drives head-to-tail associations that can be altered to attain diverse levels of compacting within cell clusters. We propose this may represent a conserved mechanism of aggregation across the large family of SAATs and may contribute to adaptation to different ecological niches.

## Results

### Ag43 variants promote different levels of bacterial aggregation

**Fig. 1:**
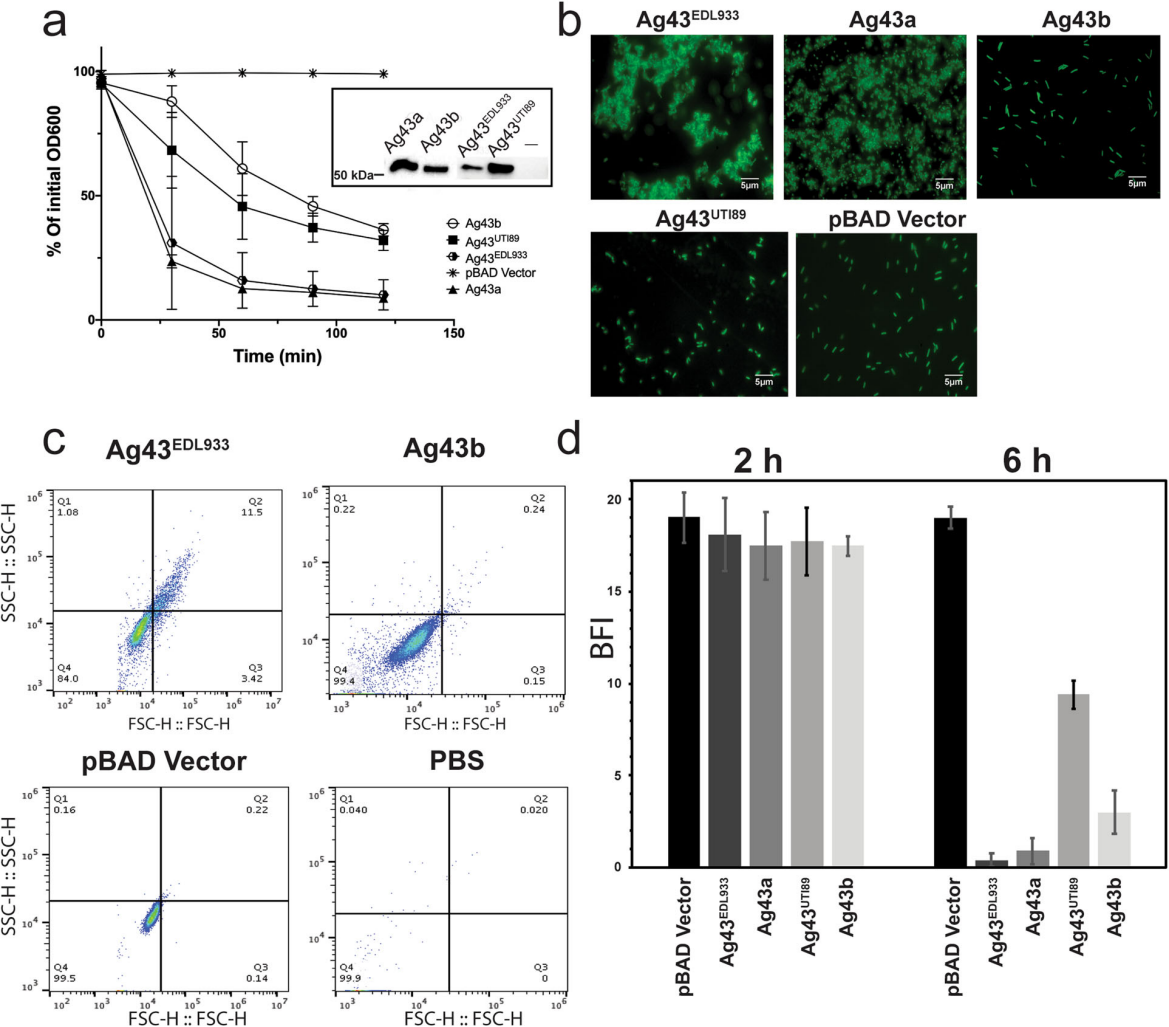
Aggregation kinetics of *E. coli* expressing the different Ag43 variants. **a** MS528 expressing Ag43a, Ag43b, Ag43^UTI89^ or Ag43^EDL933^ were left to sediment for 2 h with OD_600_ measurements taken at 30 min intervals. Experiments were performed in triplicate using MS528 harbouring pBAD/Myc-HisA as a negative control. Ag43 protein production at the bacterial cell surface was examined using western blot analysis (Ag43a and Ag43b production was detected using rabbit polyclonal serum raised against purified α^43a^, and Ag43^UTI89^ and Ag43^EDL933^ production was detected with rabbit polyclonal serum raised against α^43_UTI89^/α^43_EDL933^). **b** Representative images of GFP-positive OS56 cells harbouring the same Ag43 plasmids acquired using a Zeiss Axioplan 2 fluorescence microscope, at an early 30 min timepoint. Scale bar is 5 µm. **c** Forward vs. side scatter plot for flow cytometry performed on OS56 harbouring plasmids encoding Ag43^EDL933^ or Ag43b, control MS528 cells harbouring pBAD/Myc-HisA or PBS. These cells were induced with arabinose and analysed at exponential growth phase. **d** Results from the biofilm ring microtitre plate assay using MS528 expressing Ag43a, Ag43b, Ag43^UTI89^ or Ag43^EDL933^. Measurements are shown after 2 and 6 h of sessile growth. Results are expressed as biofilm formation index (BFI), where lower values indicate increased biofilm formation; a BFI ≤ 2 is indicative of strong biofilm production. Error bars correspond to the standard deviation calculated from three independent experiments.

We first examined the aggregation properties of a set of Ag43 variants from different *E. coli* pathogens that capture some of the phylogenetic diversity in the Ag43 family^22^. Specifically, we focused on Ag43 from EHEC EDL933 (Ag43^EDL933^) and UPEC UTI89 (Ag43^UTI89^), which belong to the C2 and C4 Ag43 phylogenetic classes, respectively^17,22,32^. We also selected Ag43b from UPEC CFT073, which belongs to the C3 group^14,22^; Ag43a, which we have characterised previously and also belongs to the C3 group^13,22^, served as a control. Each gene was PCR amplified and cloned into the expression vector pBAD/Myc-HisA to produce the full-length proteins with no tags. The resultant plasmids were transformed into the *E. coli fim agn43* null strain MS528^33^, and their ability to promote bacterial aggregation was compared using a sedimentation assay that measured the settling kinetics of standing cultures over time (Fig. 1a). Recombinant MS528 strains harbouring each of the different Ag43 variants were observed to auto aggregate. However, there were clear differences in the aggregation kinetics; while cells expressing Ag43^EDL933^ or Ag43a sedimented rapidly, cells expressing Ag43^UTI89^ or Ag43b exhibited a slower sedimentation profile (Fig. 1a). These differences were not due to major differences in the expression of the Ag43 proteins on the cell surface, as demonstrated by western blot analysis of heat-released proteins (Fig. 1a, inset) and whole-cell ELISA experiments using MS528 expressing the different Ag43 proteins (Supplementary Fig. 1). The differences were apparent within the early stage of bacterial aggregation (30 min) onwards, with fluorescence microscopy of the *agn43*-negative *gfp*-positive *E. coli* K-12 strain OS56 harbouring the different Ag43 variants revealing larger aggregates for Ag43^EDL933^ and Ag43a compared to Ag43^UTI89^ and Ag43b (Fig. 1b). Using flow cytometry, we compared Ag43^EDL933^ to Ag43b on either side of the sedimentation range, again confirming increased aggregation mediated by Ag43^EDL933^ (Fig. 1c). As a complementary measure of aggregation and biofilm formation kinetics we used the biofilm ring test^34^, an assay where the movement of microbeads in a microplate well is blocked in the course of biofilm formation. In this assay we used the recombinant MS528 *E. coli* strain expressing the different Ag43 variants, respectively. In this assay, the most marked difference occurred after six hours of *E. coli* sessile growth, whereby cells expressing Ag43a or Ag43^EDL933^ cells completely blocked the microbead movement compared to cells expressing Ag43b or Ag43^UTI89^ (Fig. 1d). This is in agreement with the sedimentation assay, where *E. coli* cells expressing Ag43a and Ag43^EDL933^ deposited more rapidly due to the formation of larger aggregates, which in turn resulted in more rapid biofilm formation compared to Ag43^UTI89^ and Ag43b.

### Different Ag43 variants adopt the L-shaped β-helical structure

**Fig. 2:**
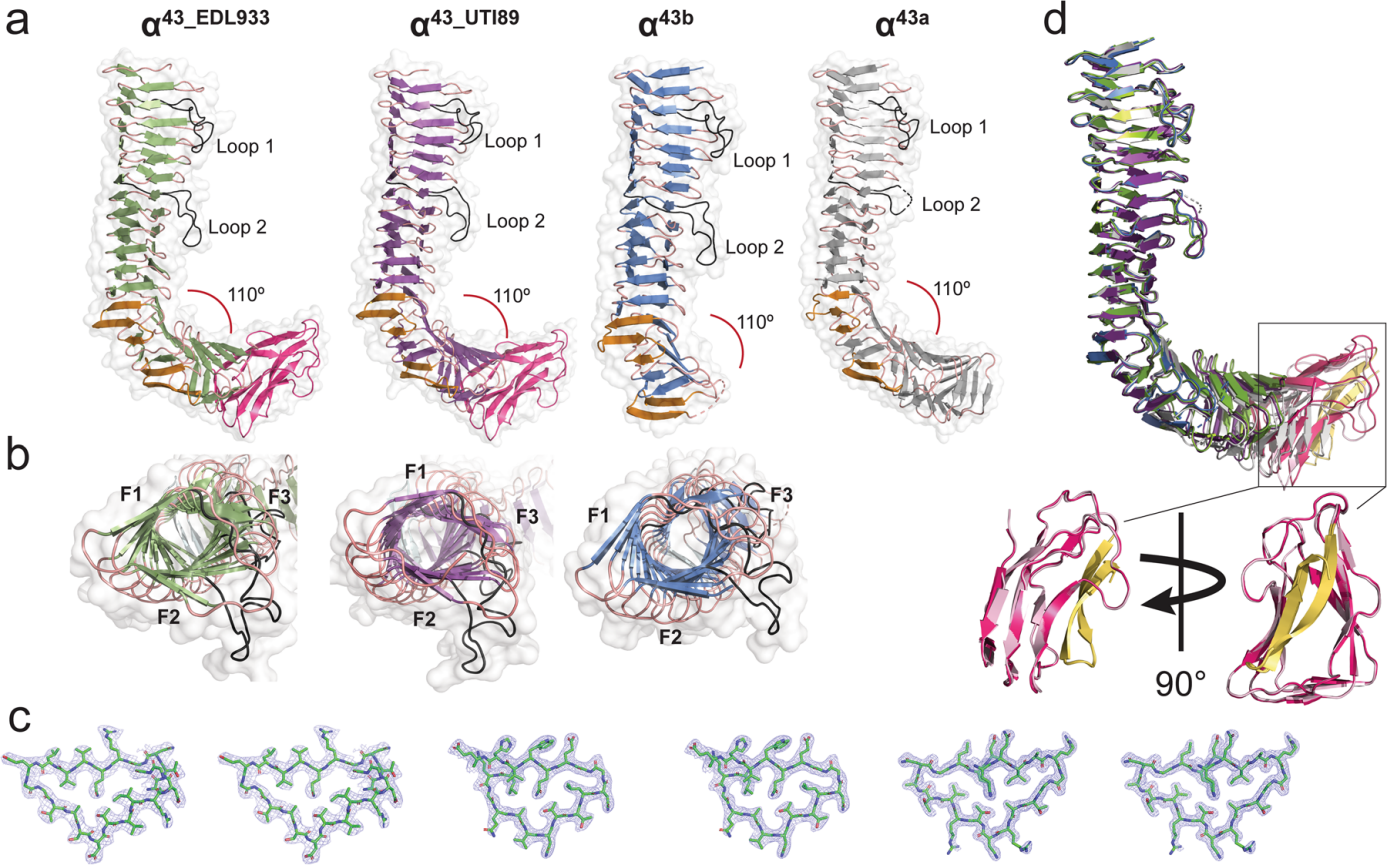
Structure of the α-domain of Ag43^EDL_933^ (α^43_EDL933^), Ag43^UTI89^ (α^43_UTI89^), and Ag43b (α^43b^). **a**, **b** Side and top cartoon representations with β-strands in green, purple and blue, respectively. Turns are coloured in salmon and F1–F3 represent the three faces of the three-stranded β-helix. The two loops (loop 1 and loop 2) that protrude from the β-helices are shown in black, while the four β-hairpins appear in light orange. The AC domain of both α^43_EDL933^ and α^43_UTI89^ are coloured in hot pink. Side view of α^43a^ (PDB: 4KH3), depicted in grey is shown for comparison^13^. **c** Stereo view of the 2Fo−Fc electron density maps contoured at 1*σ* of a cross-section of α^43_EDL933^ (residues T^65^-D^84^), α^43_UTI89^ (residues L^90^-E^108^) and α^43b^ (residues I^52^-D^70^) β-helices. **d** Superimposition of α^43_EDL933^, α^43_UTI89^ and α^43b^ on that of α^43a^, with α^43a^ depicted in grey. The AC domain is displayed in hot pink and light pink for α^43_EDL933^ and α^43_UTI89^ respectively, with the β-hairpin motifs yellow. RMSD values for all superpositions are shown in Supplementary Table S1.

The different functional properties of Ag43 variants prompted us to characterise their atomic structures, noting that the passenger domains of Ag43^EDL933^, Ag43^UTI89^ and Ag43b share 66%, 62% and 86% sequence identity, respectively, with Ag43a (Supplementary Fig. 2). All α-domain proteins were recombinantly expressed in the cytoplasm. The crystal structures of α^43_UTI89^ and α^43b^ were solved by molecular replacement using the previously determined structure of α^43a^ (PDB: 4KH3)^13^ as a search model (Fig. 2). The structure of α^43_EDL933^ was solved using α^43_UTI89^ as a reference (Fig. 2). Crystals of α^43_UTI89^ and α^43b^ belong to the *C*2 and *P*2_1_22_1_ space groups, respectively, and each contained one molecule per asymmetric unit (Table 1). The structures of α^43_UTI89^ and α^43b^ were refined to 2.4 and 2.08 Å resolution and final *R*_factor_ values of 0.16 and 0.17 (*R*_free_ 0.21 and 0.20), respectively (Table 1).

**Table 1:** X-ray crystallography data collection and statistics.

Data collection	α^43_EDL933^	α^43_UTI89^	α^43b^
PDB code	7KOH	7KO9	7KOB
Wavelength (Å)	0.9537	0.9537	0.9537
Resolution range (Å)	50.83–2.98 (3.03–2.98)	39.42–2.43 (2.52–2.43)	36.67–2.08 (2.14–2.08)
Space group	P2_1_2_1_2_1_	C2	P2_1_22_1_
Unit-cell parameters (Å, °)	97.55, 178.66, 246.23 90, 90, 90	129.28, 132.82, 47.25 90, 94.13, 90	32.78, 70.89, 171.42 90, 90, 90
Unique reflections^a^	86,188 (4118)	28,776 (3008)	24,576 (1769)
Multiplicity^a^	6.4 (6.4)	3.7 (3.8)	6.2 (5.9)
Completeness (%)^a^	97.3 (92.2)	96.3 (96.1)	98.4 (94.4)
Mean *I*/*σ*(*I*)^a^	4.6 (0.9)	9.9 (2.0)	8.3 (2.3)
*R*_meas_	0.52 (2.79)	0.10 (0.81)	0.15 (0.83)
*R*_pim_^a^	0.19 (1.03)	0.05 (0.42)	0.06 (0.33)
CC_(1/2)_^a^	0.94 (0.3)	0.99 (0.7)	0.99 (0.7)
Reflections used in refinement	86,090 (8042)	28,758 (2858)	24,511 (2285)
Reflections used for R-free	4222 (387)	1325 (119)	1285 (132)
R-work/R-free^a^	0.21(0.32)/0.25(0.36)	0.16(0.25)/0.21(0.28)	0.17(0.24)/0.20(0.26)
RMS (bonds)	0.01	0.007	0.007
RMS (angles)	1.19	0.93	0.85
Ramachandran favoured (%)	95.28	98.5	97.48
Ramachandran allowed (%)	4.62	1.5	2.52
Ramachandran outliers (%)	0.1	0	0
Rotamer outliers (%)	0.06	0	0
Clashcore (%)	8.25	5.39	4.78
Average *B*-factor (Å^2^)	45.88	50.1	26.71
Macromolecules	45.85	49.88	26.02
Ligands	58.28	65.04	63.36
Solvent	32.78	49.97	30.62
Number of TLS groups	17	4	5

^a^Information in parenthesis corresponds to values from the highest resolution shell.

α^43_EDL933^ crystals belonged to the space group *P*2_1_2_1_2_1_, diffracting to 2.98 Å and contained four molecules per asymmetric unit. Following several steps of model building and refinement the structure of α^43_EDL933^ was refined to a *R*_factor_ of 0.21 (*R*_free_ of 0.25) (Table 1). Pairwise comparison of the four symmetrically independent monomers gave an r.m.s.d. value for all Cα atoms of around 0.6 Å. As there were no significant differences observed between the molecules in the asymmetric unit, all subsequent structural comparisons were performed using the best-defined monomer.

Similar to α^43a^ ^13^, the α-domain of all three Ag43 variants folds into an L-shaped right-handed three-stranded β-helix, with each turn of the β-spine comprising three β-strands linked by loop regions (Fig. 2a). In all cases, the long arm of the L-shape is formed by 13 turns, with the bent region formed by a pair of β-hairpin motifs on either side of three β-helix rungs, followed by a shorter C-terminal β-helical domain. We predict the full-length α^43b^ structure resembles the L-shaped α^43a^ since the crystallised truncated form has the two bending β-hairpin motifs and α^43b^ shares 94.5% sequence identity with α^43a^ at the C-terminal end of the β-helix (Supplementary Fig. 2). α^43_UTI89^ and α^43_EDL933^ are shorter than α^43a^ and α^43b^; sequence alignment of these proteins showed that the former proteins are reduced in size by 69 amino acids in the α-domain (Supplementary Fig. 2), which results in the C-terminal β-helix domain consisting of four rungs instead of seven in α^43a^ (Fig. 2a, Supplementary Fig. 3). Although α^43a^ and α^43b^ are similar in size, the α^43b^ crystallised in this work is truncated and lacks the bottom section of the L-shaped β-helix (Fig. 2a). The α^43b^ truncation occurred during recombinant protein purification with cleavage occurring at residue 415, possibly by a protease mechanism that remains to be defined. In all proteins, two loops protrude from the β-helix between rungs 2 and 3 (loop 1, 11–14 residues) and rungs 7 and 8 (loop 2, 11 residues). The residues in these loops include negatively charged residues which create acidic patches that protrude from the β-spine (Supplementary Fig. 4). No functional role has been attributed to these protruding loops to date^13^.

### α^43_UTI89^ and α^43_EDL933^ structures incorporate the autochaperone domain

During translocation to the bacterial cell surface, the functional α-domain of Ag43 is cleaved but remains attached to the C-terminal β-domain via non-covalent interactions^13,19^. The specific cleavage site varies among Ag43 variants but typically localises between the β-helix and the predicted autochaperone (AC) domain^35^. The α^43_UTI89^ and α^43_EDL933^ structures in this work correspond to the uncleaved α-domains.

Present within the structures of α^43_UTI89^ and α^43_EDL933^, their AC regions consist of three β-strand rungs capped by a β-hairpin motif (Fig. 2d). This region shows structural similarities with previously characterised AT domains; where it can be superimposed with r.m.s.d values ranging between 1.50 and 4.37 Å with the AC domain from EspP (PDB: 3SZE), IcsA (PDB: 3ML3), p69 (PDB: 1DAB), Hap (PDB: 3SYJ) and Hbp (PDB: 1WXR)^36–41^ (Supplementary Table 1). Previous studies have shown the AC is involved in α-domain folding^30,42–44^. In the α^43_UTI89^ and α^43_EDL933^ structures the main β-helix and AC domain is covalently linked via an extended 18 amino acid loop. This loop is mostly undefined in the electron density maps of α^43_UTI89^ (434–451) and α^43_EDL933^ (435–452), possibly due to its flexible properties.

### Ag43^UTI89^ and Ag43b self-associate via an interface with fewer interactions

**Fig. 3:**
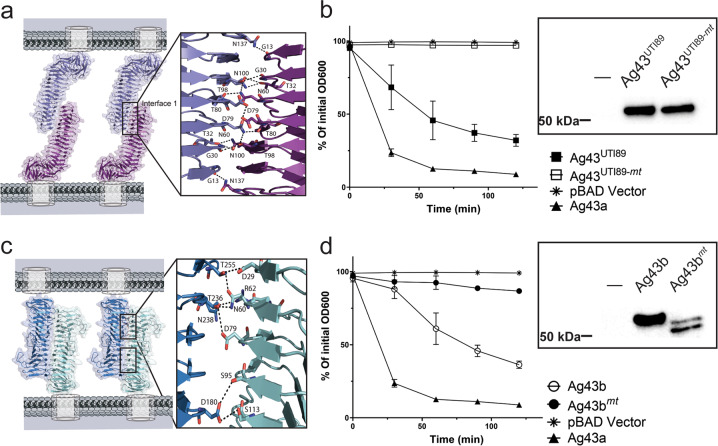
Ag43 interfaces with reduced self-association interactions. **a** Self-association of α^43_UTI89^ via a single interface consisting of 13 hydrogen bonds [G13-N137, G30-T98, T32-N100 (two hydrogen bonds), N60-T98, N60-T80, D79-D79, T80-N60, T98-G30, T98-N60, N100-T32 (two hydrogen bonds) and N137-G13]. **b** Confirmation of the α^43_UTI89^ self-association interface shown by the aggregation profile of MS528 expressing the interface mutant Ag43^UTI89-*mt*^ (T32G, N60G, D79G, T80G, T98G, N100G, N137G), WT Ag43^UTI89^, WT Ag43a and the control pBAD/Myc-HisA plasmid. The expression of the heat-released Ag43 α-domain was examined by western blot analysis (inset). **c** Self-association of α^43b^ via a double interface with each consisting of seven hydrogen bonds [D29-T255, N60-T255, R62-N238, D79-T236, S95-D180, S113-D180 and N60-T236]. **d** Confirmation of the α^43b^ self-association interface shown by the aggregation profiles of MS528 expressing the interface mutant Ag43b^*mt*^ (D29G, N60G, R62G, D79G and S95G), WT Ag43b, WT Ag43a and the control pBAD/Myc-HisA plasmid. The expression of the heat-released Ag43 α-domain was examined by western blot analysis (inset). Error bars correspond to the standard deviation calculated from three independent experiments.

**Fig. 4:**
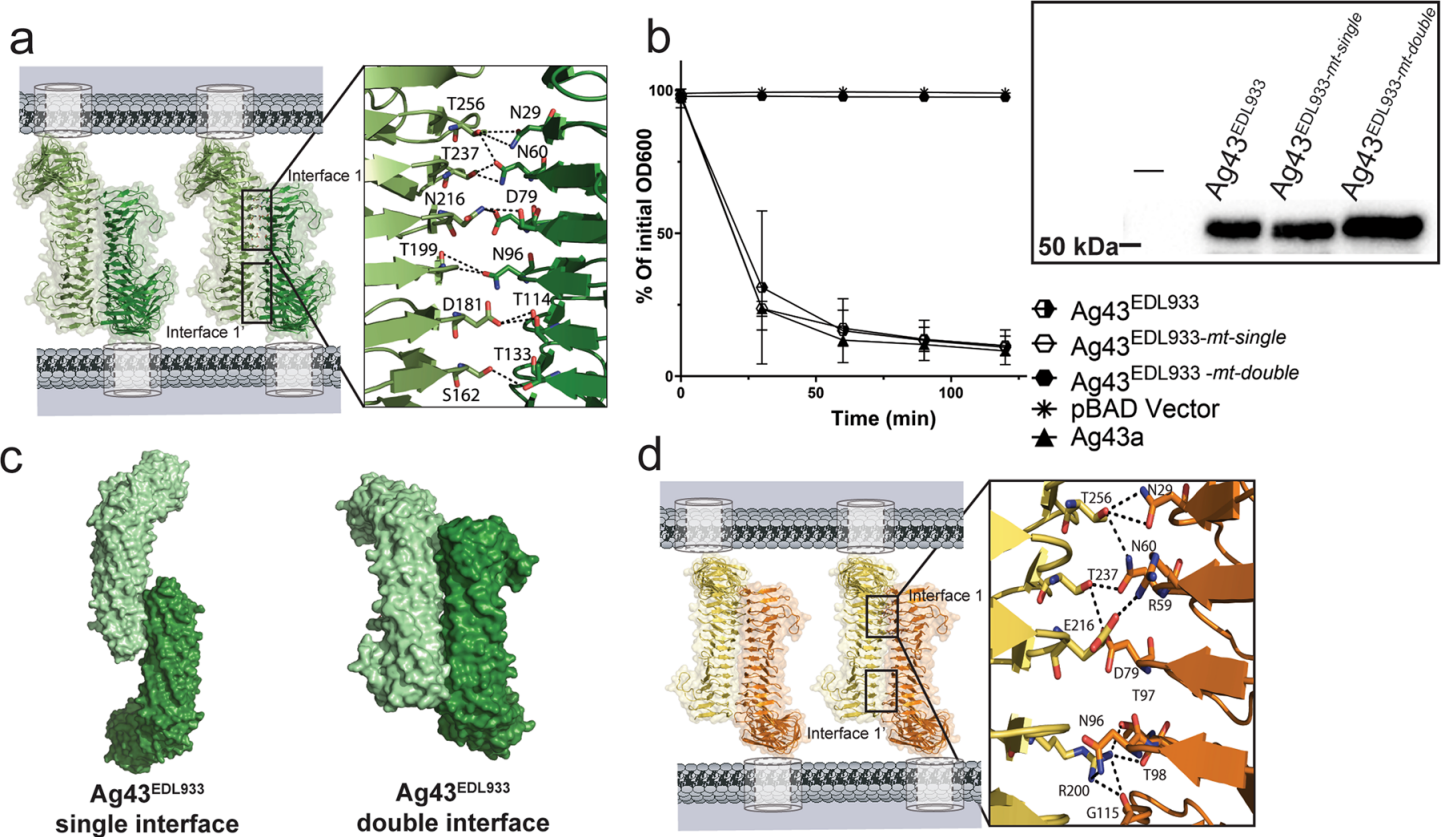
Ag43 interfaces with extensive self-association interactions. **a** Double self-association interface of α^43_EDL933^, with each interface consisting of 12 hydrogen bonds [D181-T114 (2 H-bonds), T256-N29 (2 H-bonds), S162–T133, N216-D79, N216-S78, T237-N60 (2 H-bonds) T256-N60, N96-T199 (2 H-bonds)]. **b** Confirmation of the α^43_EDL933^ double interface was shown by the loss of aggregation for the double interface mutant Ag43^EDL933-*mt-double*^ (T199G and T256G). In contrast, mutation of the predicted single interface Ag43^EDL933-*mt-single*^ (T15G, T32G, N60G, D79G, T98G, N100G, N119G and N138G) showed no effect on aggregation. Aggregation assays examined MS528 expressing WT Ag43^EDL933^, WT Ag43a and containing the control pBAD/Myc-HisA plasmid. The expression of the heat-released Ag43 α-domain was examined by western blot analysis (inset). Error bars correspond to the standard deviation calculated from three independent experiments. **c** A depiction of the two alternative modes of α^43_EDL933^ self-association using a single or double interface. **d** Self-association of α^43a^ with each interface comprising 9 hydrogen bonds and a salt bridge [N29-T256 (2 H-bonds), N60-T256, N60-T237, D79-T237, N96-R200, T97-R200, T98-R200, G115-R200 and R59-E216 (salt bridge)]^13^.

Ag43a from CFT073 drives bacterial aggregation by homotypic interactions between α^43a^ molecules from neighbouring cells in a head-to-tail conformation^13^. To investigate whether this mechanism of self-association is conserved in Ag43^UTI89^, which imparts a reduced aggregation phenotype, we first examined its oligomerisation in the crystal lattice. Generation of crystallographically related molecules of α^43_UTI89^ (i.e. symmetrically related molecules in the crystal lattice) revealed a head-to-tail dimer repeated along the crystal, which resembled the α^43a^–α^43a^ functional dimer (Fig. 4d)^13^. However, while α^43a^ trans-dimers are stabilised by two interfaces consisting of a total of 18 hydrogen bonds and two salt bridges, α^43_UTI89^ dimers are formed via interactions from a single interface comprising 13 hydrogen bonds (Fig. 3a, Table 2). To test if this α^43_UTI89^ crystallographic interface was indeed involved in mediating bacterial aggregation, we constructed a mutant with seven amino acid substitutions (Ag43^UTI89-*mt*^: T32G, N60G, D79G, T80G, T98G, N100G, N137G; mutations to glycine were adopted as glycine residues are enriched in the loop regions of Ag43 β-helices; Supplementary Fig. 2). Plasmid constructs harbouring full length Ag43^UTI89^ (pBAD::Ag43^UTI89^) or Ag43^UTI89-*mt*^ (pBAD::Ag43^UTI89-*mt*^), were transformed into MS528 and tested in cell-aggregation assays. Modification of the single interface in Ag43^UTI89^ completely abolished the ability of this protein to promote aggregation (Fig. 3b), confirming the self-association interface observed in the crystal lattice. This loss of function was not due to lack of expression of Ag43^UTI89^ on the bacterial surface as shown by western blot analysis of heat-released proteins (Fig. 3b, inset) and whole-cell ELISA experiments using MS528 expressing native and mutant Ag43^UTI89^ (Supplementary Fig. 1).

**Table 2:** List of residues involved in the Ag43 self-association interfaces.

Dimers	Interface residue interactions	# of interactions
α^43_UTI89^–α^43_UTI89^	H-bonds: G13-N137, G30-N100, T32-N100 (2 H-bonds), N60-N100, N60-T98, D79-D79, T80-N60, T98-N60, T98-G30, N100-T32 (2 H-bonds), N137-G13	13 Hydrogen bonds
α^43b^–α^43b^	H-bonds: D29-T255, N60-T255, N60-T236, R62-N238, D79-T236, S95-D180, S113-D180 (in each interface)	14 Hydrogen bonds
α^43_EDL933^–α^43_EDL933^	H-bonds: D181-T114 (2 H-bonds), T256-N29 (2 H-bonds), S162-T133, N216-D79, N216-S78, T237-N60 (2 H-bonds) T256-N60, N96-T199 (2 H-bonds) (in each interface)	24 Hydrogen bonds
α^43a^–α^43a^ ^13^	H-bonds: N29-T256 (2 H-bonds), N60-T256, N60-T237, D79-T237, N96-R200, T97-R200, T98-R200, G115-R200 (in each interface) Salt bridge: R59 - E216 (in each interface)	18 Hydrogen bonds 2 Salt bridges

Although Ag43a and Ag43b share 86% sequence identity in their respective α-domains, these proteins differ significantly in their aggregation profiles (Fig. 1a)^14^. Unlike α^43a^, α^43b^ did not crystallise in its dimeric form. However, given the similarity between α^43a^ and α^43b^, we superimposed two α^43b^ structures on the α^43a^–α^43a^ functional dimer^13^ to infer the potential α^43b^ dimerisation interface, which consisted of two interfaces encompassing a total of 14 hydrogen bonds (Fig. 3c, Table 2). To confirm if this interface was indeed responsible for the aggregation phenotype of Ag43b, we mutated residues D29, N60, R62, D79 and S95 to glycine (Ag43b^*mt*^) and tested an MS528 strain expressing this mutant protein in aggregation assays. Substitution of these five interface residues completely abolished the ability of Ag43b to mediate cell–cell aggregation compared with Ag43b WT, confirming the predicted mode of self-association (Fig. 3d). Western-blot analysis of the heat released Ag43b^*mt*^ showed some level of degradation compared to native Ag43b, possibly caused by the heat treatment of the mutant protein; however, whole-cell ELISA experiments revealed a similar level of surface expression of both native and mutant Ag43b, which are comparable to Ag43a wild-type and Ag43a interface mutant^13^ expression (Supplementary Fig. 1).

### Ag43^EDL933^ self-associates through a double interface with extensive interactions

The α^43_EDL933^ crystals with four molecules in the asymmetric unit revealed complex non-crystallographic and crystallographic oligomers, however, none of these resembled previously defined functional α^43^ dimers where the β-helical molecules coil around each other in trans-configuration^13^ (Supplementary Fig. 5). To define the self-association interface that leads to Ag43^EDL933^ bacterial clumping, α^43_EDL933^ molecules were superimposed onto α^43a^ dimers, stabilised by two interfaces^13^ (Fig. 4c), and onto α^43_UTI89^ dimers with a single interacting interface (Fig. 4c; Supplementary Fig. 6). All possible interactions at the interfaces of both models were predicted by exploring the different rotamer conformations of the residues mapping near the putative interfaces. The first superposition predicted an α^43_EDL933^ functional dimer stabilised by two interfaces, each consisting of 12 hydrogen bonds (Fig. 4a, Table 2). Conversely, when the α^43_UTI89^ functional dimer was used as the scaffold, α^43_EDL933^ molecules were predicted to self-associate via one interface also encompassing 24 hydrogen bonds (Supplementary Fig. 6). We designed two sets of mutants to discern between an Ag43^EDL933^ double or single interface self-association. To test for the single interface, we mutated T15, T32, N60, D79, T98, N100, N119 and N138 to glycine residues (Ag43^EDL933-*mt-single*^). We generated a second mutant where we mutated T199 and T256 to glycine at the base of α^43_EDL-933^ (Ag43^EDL933-*mt-double*^), mapping far from the predicted single interface, and responsible for 10 out of the total predicted 24 hydrogen bonds stabilising the putative Ag43^EDL933^ double interface dimer. Examination of MS528 strains expressing these mutants in cell-aggregation assays showed that while modification of the predicted Ag43^EDL933^ single interface did not alter its aggregative function, mutation of two residues in the double interface completely abolished Ag43^EDL933^ mediated cell aggregation (Fig. 4b). This was not due to differences in the expression of the mutant Ag43^EDL933^ proteins on the cell surface, as confirmed by western blot analysis of heat-released proteins (Fig. 4b, Inset) and whole-cell ELISA experiments (Supplementary Fig. 1). Hence, we confirmed that α^43_EDL933^ interacts via an extensive double interface. Although both dimers are possible, the double interface dimer results in a 550 Å^2^ buried surface area, compared to 260 Å^2^ for the single interface dimer, suggesting that interactions such as van der Waals contacts are also important for dictating the preference for the double interface dimer.

### The oligomeric state of Ag43 variants in solution is consistent with their functional properties

**Fig. 5:**
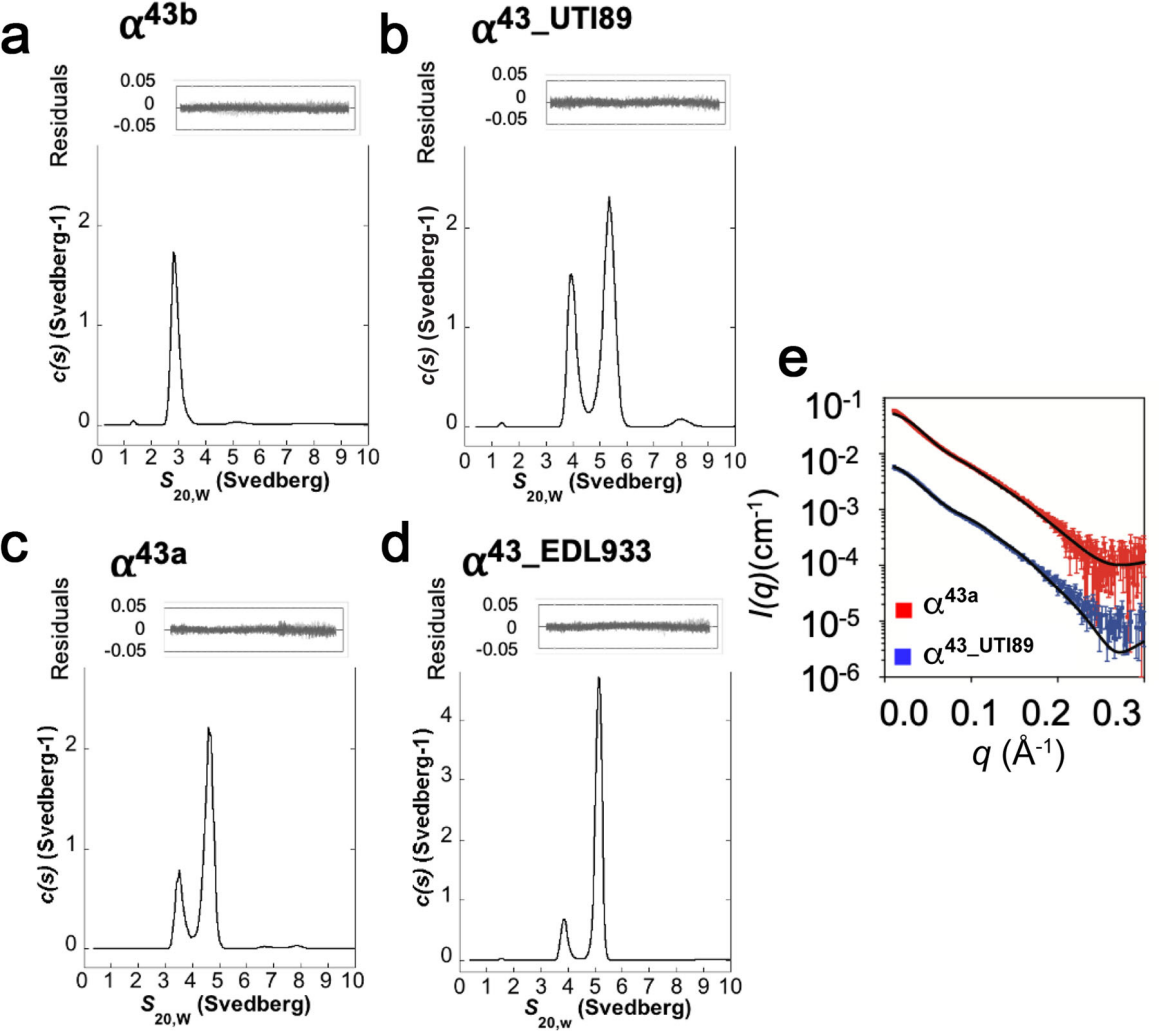
Oligomeric state of Ag43 variants in solution. Representative analytical ultracentrifugation (AUC) sedimentation velocity analysis of the Ag43 variants at 2.2 mg/mL with *c*(*s*) plotted as a function of *s*_20,w_ (Svedberg). Both α^43a^ **c** and α^43_EDL933^ **d** showed a much higher proportion of dimer (4.7 and 5.2 S) than monomer (3.5 and 3.8 S). In comparison α^43_UTI89^ **b** revealed a higher proportion of monomer (3.9 S) than dimer (5.4 S), whereas α^43b^ **a** showed only a monomeric species (2.8 S). Residuals resulting from the *c*(*s*) distribution fits are shown above. Small-angle X-ray scattering measurements of α^43a^ (red) and α^43_UTI89^ (blue) in solution **e**. Model scattering curves are overlayed on the experimental data (solid black line) and have been calculated as a linear combination of monomer and dimer scattering curves. The model scattering curves are consistent with the experimental data (*χ*^2^(α^43a^) = 22.9; *χ*^2^(α^43_UTI89^) = 8.8).

To examine the self-association properties of Ag43 variants in solution, we analysed recombinantly expressed and purified proteins by analytical ultracentrifugation sedimentation velocity and small angle scattering experiments. Sedimentation-coefficient distribution (*c*(*s*)) analysis of α^43a^ (Fig. 5c) and α^43_EDL933^ (Fig. 5d) at 2.2 mg ml^−1^, showed that these two proteins exist primarily as dimeric species with standardised sedimentation coefficients (*s*_20,w_) of 4.7 and 5.2 S, with lower proportions of a monomeric species at 3.5 and 3.8 S, respectively. Conversely, at the same concentration α^43_UTI89^ (Fig. 5b) and α^43b^ (Fig. 5a) showed higher proportions of monomeric species at 3.9 and 2.8 S, respectively. Both α^43_UTI89^ and α^43_EDL933^ have two-fold higher extinction coefficients than both Ag43a and Ag43b, and showed correspondingly higher *c*(*s*) values. This data supports the capacity of the Ag43 variants to form dimers in solution, consistent with their ability to promote aggregation. Importantly, the finding that both α^43a^ and α^43_EDL933^ form higher proportions of dimers compared to α^43b^ and α^43_UTI89^ at the same concentration correlates with their faster aggregation kinetics (α^43b^ dimerisation was only apparent when the sample was concentrated above 30 mg/ml prior to AUC analysis; Supplementary Fig. 7). Additionally, the frictional ratios (*f*/*f*_0_) for these experiments ranged from 1.4 to 1.8, confirming the extended shape of both the monomers and the *trans*-dimers.

We further investigated the solution properties of the two different Ag43 dimer conformations represented by α^43a^ and α^43_UTI89^ using small-angle X-ray scattering (SAXS) (Supplementary Table 2). In both cases, analysis of the SAXS data found the two Ag43 variants to be mixtures of different oligomeric states. To understand the way in which these proteins oligomerise, and to quantify the amount of monomer and dimer present in solution, scattering profiles of the monomeric and dimeric forms were calculated from the α^43a^ and α^43_UTI89^ crystal structures, which represent a more extensive double interface and smaller single interface, respectively. The experimental SAXS data were then fit as a linear combination of the model monomer and dimer curves. In both cases, the quality of the fit to the data was good (Fig. 5e), providing evidence that the dimerisation occurs in the manner displayed in the crystal structures for both proteins. Further, the analysis indicated that at 1.2 mg mL^−1^, the concentration used for SAXS, a higher proportion of the protein was present in the dimeric form for α^43a^ compared to α^43_UTI89^, which is consistent with the findings from the analytical ultracentrifugation experiments that show α^43a^ has a higher propensity to form dimers compared to α^43_UTI89^.

## Discussion

The SAAT subgroup of ATs are major determinants of bacterial aggregation and biofilm formation^9^. In an aggregated state, bacteria are more resistant to multiple stresses, including those induced by antibiotics and biocides. The protection offered by bacterial aggregation and biofilm formation is an important contributor to the development of antibiotic resistance and chronic infections, therefore understanding these processes will inform the development of new approaches to combat the increasing threat posed by multidrug-resistant bacteria. While the biology of aggregating ATs has been well investigated, understanding the molecular mechanisms that drive bacterial aggregation has been hampered by the lack of atomic detail for the majority of AT proteins that mediate this function.

Ag43 is one of the best-characterised SAATs in *E. coli*, expressed in commensals as well as a wide variety of pathogens including UPEC, EHEC and EPEC strains^9,14,17,45–48^. This major phase variable outer membrane protein is a prototype adhesin for bacterial aggregation studies, and also mediates biofilm production, including the formation of biofilm like intracellular communities (IBCs) and multispecies biofilms, as well as enhancing colonisation and persistence in the bladder^14,33,47,49^. Phylogenetic network analysis focusing on Ag43 functional domains classified this family of proteins into four major groups (C1–C4) based on sequence variation, with variable aggregation properties observed among individual proteins across and within different groups^22^. In the current study, we have focused on a set of Ag43 variants from strains representing two clinically important *E. coli* pathotypes: EDL933 (EHEC) as well as UTI89 and CFT073 (UPEC). Our characterisation of the cell aggregation phenotypes of Ag43^EDL933^, Ag43^UTI89^ and Ag43b compared to Ag43a, are largely consistent with previous studies^22^, but show some further variations. Ag43^EDL933^ (C2) shows a strong aggregation phenotype that is also shared by Ag43a (C3). However, Ag43b, which is also from group C3, mediated reduced aggregation, thus demonstrating inter-group functional variability. Our data show that this difference in aggregation between Ag43a and Ag43b is based on amino acid variation in their interacting interfaces. Like Ag43b, Ag43^UTI89^ (C4) also mediated reduced aggregation. The aggregation properties of these variants were consistent with differences in bacterial clumping as shown by fluorescence microscopy and flow cytometry, with Ag43^EDL933^ showing strongest aggregation in these assays.

To define the mechanistic detail underlying the functional differences observed among the Ag43 variants we determined their crystal structures. Our previous studies on the functional domain of UPEC CFT073 Ag43a (α^43a^) revealed a distinct curved β-helical architecture and identified that trans-association of Ag43a β-helices from neighbouring cells leads to bacterial clumping^13^. In this study, we determined the structure of a further three Ag43 functional domains (α^43_EDL933^, α^43_UTI89^ and α^43b^). Of significance is the finding that despite their sequence identity, which ranged from 62% to 87%, all of the characterised Ag43 variants retain the L-shape bend, which we previously showed was required for Ag43a bacterial aggregation^13^. However, this L-shape is not present in other AT adhesins that directly bind host surfaces such as UpaB^50^ and pertactin P.69^36^. Thus, it is possible that the L-shape bend may only be required for Ag43-mediated aggregation. It remains to be determined how widespread the L-shape architecture is among the SAATs and other functionally related ATs. To date, the only other SAAT structure is of TibA from enterotoxigenic *E. coli*, however, this structure only encompasses an N-terminal portion of the passenger domain^51^.

Through structural and mutagenesis studies we showed that all Ag43 variants examined in this study self-associate in a head-to-tail manner, with the distinct aggregation dynamics observed for each variant determined by its specific inter-protein interactions. For example, the rapid aggregation phenotype displayed by Ag43^EDL933^, which mimics that of Ag43a, results from strong α^43_EDL933^–α^43_EDL933^ homotypic interactions stabilised by two interfaces with a total of 24 hydrogen bonds (α^43a^ dimers are also stabilised by two interfaces with a total of 18 H-bonds and two salt bridges)^13^. Conversely, the reduced aggregation phenotype of Ag43^UTI89^ is a direct result of the weaker α^43_UTI89^ dimers, which are only stabilised by a single interface encompassing 13 hydrogen bonds. It appears that the inability of α^43_UTI89^ to form a double interface may be due to the presence of long sidechains on residues such as R161 or D133 in the F2–F3 loops mapping in the lower part of the β-helix, which in a head-to-tail association via a double interface would result in steric clashes between interacting proteins (Supplementary Fig. 8), although we cannot rule out other amino acids that might also interfere with this interaction. Although α^43b^–α^43b^ trans-dimers are stabilised by a double-interface, this interface consists of only 14 hydrogen bonds. This reduced bonding network results in Ag43b-expressing cells displaying a reduced aggregation profile comparable to that of Ag43^UTI89^. The differing strengths and configurations of these Ag43 self-associations interpreted from the crystal structures were confirmed in solution using both AUC and SAXS analysis. Consistent with the aggregation phenotypes, both α^43_EDL933^ and α^43a^ showed a higher propensity for self-association compared to α^43_UTI89^ and α^43b^.

Our study shows how bacterial adhesins such as Ag43 can acquire simple adaptations to modify their self-association mechanisms. The introduction of additional polar or charged amino acids within the Ag43 interaction interface increases affinity and aggregation kinetics, while the presence of amino acids opposing in nature perturbs self-association thereby decreasing affinity and cell compacting. Interestingly, the Ag43 variants investigated here retained a significant degree of conservation with an approximate consensus sequence of N/D, N, D, N, (X)^*n*^, T, T, at their association interfaces. This presumably allows for heterotypic interactions to take place between different Ag43 variants to influence polymicrobial biofilms between different strains of *E. coli*^13,22^. Indeed, α^43_UTI89^ with the single self-association interface is from the C4 group of Ag43 variants that have been shown to mediate trans-associations with other Ag43 variants^22^. Future areas of investigation are likely to focus on defining the self-interaction mode for Ag43 variants from C1, which was not addressed in this work, along with investigations into the level of functional variation within each Ag43 phylogroup. Currently, predicting the self-interaction of uncharacterised Ag43 proteins is challenging because of the variability of amino acid sequences within the passenger domain of these proteins. However, as our battery of empirically determined Ag43 structures and interface types increases, these predictions may become more accurate.

**Fig. 6:**
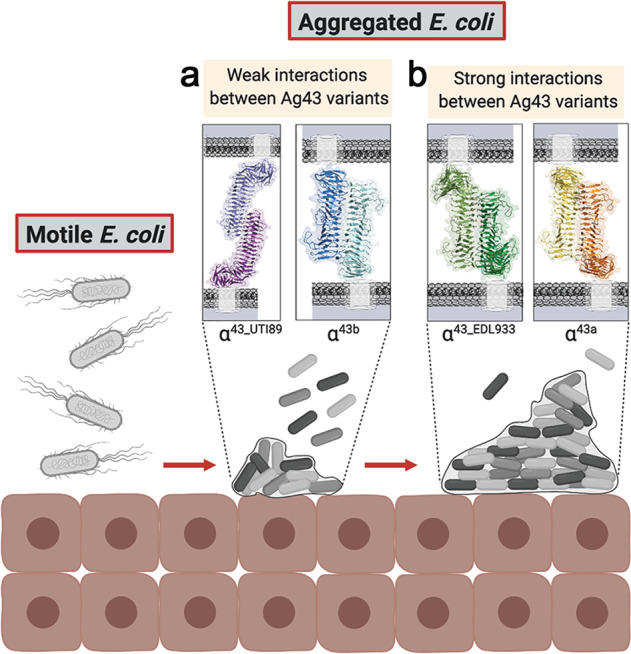
Model depicting how variation in Ag43 self-association could lead to different levels of bacterial compacting within biofilms. **a** The weaker interactions between Ag43 variants such as Ag43^UTI89^ (13 H-bonds) and Ag43b (14 H-bonds) results in reduced aggregation and biofilm formation and a greater proportion of free bacteria. **b** In contrast, the higher affinity interactions between Ag43 variants such as Ag43a (18 H-bonds and 2 salt bridges) and Ag43^EDL933^ (24 H-bonds) leads to increased aggregation and biofilm formation. These variations are adaptations by *E. coli* to suit colonisation and persistence in different environments. This image was created with Biorender.

Ag43 mediated bacterial aggregation is a complex phenomenon. In addition to regulation of Ag43 at the transcription level by phase variation, changes in the sequence of the Ag43 adhesin can lead to different degrees of aggregation and biofilm formation (Fig. 6). Bacteria are faced with a trade-off, whereby as aggregation is increased, more protection is afforded within the biofilm, but bacterial dissemination is reduced. An important factor that can influence bacterial aggregation and biofilm formation is the micro-environment. UPEC are exposed to varying levels of flushing by urine and host antimicrobial peptides, while EHEC intestinal colonisation results in exposure to peristalsis and diverse microbiota. In some cases, *E. coli* can express more than one Ag43, such as UPEC CFT073 with Ag43a and Ag43b, creating greater versatility, with the former displaying higher aggregation properties and being more prevalent in UPEC isolates associated with recurrent infections^14,47^. The ability of some Ag43 variants to form hybrid associations with other variants also influences the formation of polymicrobial biofilms.

During bacterial infection, adhesins such as fimbriae mediate specific binding to receptors that facilitate colonisation and determine tissue tropism. However, the function of aggregation factors such as Ag43 that facilitate subsequent stacking of bacteria at the infection site is less-well defined. We posit that Ag43, and other aggregation factors could perform this stacking function, with variations in the strength of aggregation leading to altered bacterial compacting in different environments.

## Methods

### Cloning of Ag43 variants

Cloning of *agn43a* (locus tag c3655) and *agn43b* (locus tag c1273) from UPEC CFT073 into pBAD/Myc-HisA^52^ has been previously described^14^ and constructs were available in our laboratories. PCR amplification and cloning of *agn43* from UTI89 (c1139) and *agn43* (*cah*; z1211) from EDL933 into pBAD/Myc-HisA was carried out using primers UTI89_c1139a_Lic_Fw, UTI89_c1139a_Lic_Rv, EDL933_z1211a_Lic_Fw and EDL933_z1211a_Lic_Rv (Supplementary Table 3). The pBAD/Myc-HisA plasmids expressing the full length Ag43 proteins were used as the parent vectors for the construction of all mutants, namely Ag43^UTI89-*mt*^, Ag43b^*mt*^, Ag43^EDL933-mt-*single*^, Ag43^EDL933-*mt*-*double*^ (Supplementary Table 4). All mutant constructs were generated by Epoch Life Science, confirmed by sequencing and used to transform *E. coli* strain MS528^16,33^ for functional testing.

### Bacterial sedimentation assays

pBAD/Myc-HisA plasmids encoding wild-type and mutant versions of Ag43a, Ag43b, Ag43^UTI89^ and Ag43^EDL933^ were transformed into the *E. coli fim agn43* null strain MS528, which is unable to mediate cell aggregation^16^. Expression of the proteins was induced by incubation in LB media with 0.2% w/v l-arabinose for 3 h at 37 °C. Bacterial cultures were then adjusted to an optical density at 600 nm (OD_600_) of 2. A volume of 1 mL of each culture was transferred to cuvettes and left to stand at room temperature. OD_600_ measurements were taken at 30 min intervals for 2 h. All assays were performed in triplicate.

### Flow cytometry

pBAD/Myc-HisA plasmids encoding Ag43a, Ag43b, Ag43^UTI89^ and Ag43^EDL933^ were transformed into the *agn43*-negative gfp-positive *E. coli* K-12 strain OS56^14^. Each sample was washed in PBS once and suspended in equal volume of PBS. Sample dilutions in PBS were analysed in triplicate using BD Accuri C6 flow cytometer (BD Bioscience, San Diego, CA, USA) 488 nm laser for excitation and measuring emission at 530/30 nm. Readings were collected in logarithmic mode comprising at least 5000 events per sample. Data was analysed using FlowJo 10.X.7 (Tree Star).

### Heat release assays

Bacterial cultures were prepared following the same method described for sedimentation assays, which involved using the pBAD/Myc-HisA plasmids encoding wild-type and mutant versions of Ag43a, Ag43b, Ag43^UTI89^ and Ag43^EDL933^ to transform into the *E. coli fim agn43* null strain MS528^16^. Expression of the proteins was induced by incubation in LB media with 0.2% w/v l-arabinose for 3 h at 37 °C. After normalising the OD_600_ to 2, the cultures were heated to 60 °C for 2 min to release the α-domains from the cell surface into the supernatant. The heat-released samples were analysed by western blot analysis to evaluate the translocation and the production levels of both Ag43 wild-type and mutant proteins on the bacterial cell surface. Blotting was performed by blocking with 3% skim in TBST (TBS with 0.05% Tween 20), incubation with primary rabbit polyclonal antibody α^43_UTI89^/α^43_EDL933^ (1:10,000) to detect α^43_UTI89^ and or primary rabbit polyclonal antibody α^43a^ (1:10,000) to detect and α^43a^ and α^43b^, followed by incubation with secondary anti-rabbit IgG/HRP antibody (Promega, W401B, 1:10,000). Bands were detected using Bio-rad Clarity Western ECL Substrate. The uncropped and unprocessed scans of the blots are included in the Source Data file.

### Ag43 immunodetection on the bacterial cell surface

pBAD/Myc-HisA plasmids encoding wild-type and mutant versions of Ag43a, Ag43b, Ag43^UTI89^ and Ag43^EDL933^ were transformed into the *E. coli fim agn43* null strain MS528. Bacterial cultures were grown in 3 ml of LB supplemented with ampicillin (100 μg/ml) and 0.2% arabinose at 37 °C overnight. Cells were resuspended in 0.1 M sodium carbonate pH 9.5 to OD_600nm_ of 1. MaxiSorp 96-well ELISA plate (Thermo Fisher 44-2404-21) wells were coated with 100 μl of the cell suspension and incubated overnight at 4 °C. Wells were blocked with 5% skim milk in PBST (PBS with 0.05% Tween-20) for 1 h at room temperature. Antibody incubations were performed in 100 µl PBST for 1 h at room temperature. Rabbit polyclonal serum α^43a^ diluted 1:1000 and secondary antibody, α-rabbit IgG, AP conjugated (Sigma A3687) was diluted 1:10,000. All wash steps were performed three times with 250 µl PBST. The reaction was developed with 100 µl of substrate pNPP (Sigma P7998) in the absence of light and optical density was measured at 420 nm after 10 min. Three biological replicates of each strain were measured, each with four technical replicates.

### Fluorescence microscopy of bacterial aggregates

*E. coli K-12* strain OS56 harbouring pBAD/Myc-HisA plasmids encoding Ag43a, Ag43b, Ag43^UTI89^ and Ag43^EDL933^ were induced by incubation in LB media with 0.2% w/v l-arabinose for 3 h at 37 °C. Each sample was collected at 30 min for fluorescence microscopy analysis using ZEISS Research Axioplan 2 epifluorescent/light microscope (Carl Zeiss Microimaging). All assays were performed in triplicate.

### Biofilm ring test

The Biofilm Ring Test (BRT)^34,53,54^ was used to assess biofilm formation at early stages of sessile development of MS528 with pBAD/Myc-HisA plasmids encoding wild-type Ag43a, Ag43b, Ag43^UTI89^ and Ag43^EDL933^. In brief, after adjusting the OD_600_ to 0.05 with sterile LB medium containing 0.02% arabinose, paramagnetic microbeads were added and homogenised by vortexing prior to loading into 96‐well polystyrene microplates (200 μl per well, one plate per time point). Control wells were filled with microbeads and LB. After static incubation at 37 °C for 2 or 6 h (OD_600_ of 0.2 and 0.6, respectively), wells were first covered with biofilm control (BFC) contrast liquid prior to scanning before (I_0_ image) and after 1 min magnetisation (I_1_ image). Magnetisation was carried out using a BFC magnetic rack (BFC, France), a 96 block with magnets centred at the bottom of each well, whereby free microbeads are attracted to the well centre, forming a brown ring, and microbeads trapped in a biofilm are hindered and therefore unable to move to the well centre. The I_0_ and I_1_ images for each well were compared to calculate the Biofilm Formation Index (BFI)^34^; a high BFI value corresponds to a high mobility of microbeads under magnet action (absence of biofilm formation), while a low value (BFI ≤ 2) indicates a full immobilisation of the microbeads by bacterial microcolonies (no differences between the images I_0_ and I_1_)^54^. All assays were performed in triplicate.

### Expression and purification of Ag43 α-domains

The expression construct for the Ag43b α-domain (residues 55–553) was designed based on the empirically determined cleavage site between the α^43b^ and β^43b^ domains^15^. For Ag43^UTI89^ and Ag43^EDL933^, as their precise α-domain processing sites are unknown, two different constructs were generated; a shorter construct where the cleavage site was predicted based on the Ag43a processing site^15^ (Ag43^UTI89^, residues 53–482 and Ag43^EDL933^, residues 53–483); and a longer construct which included the complete passenger domain also comprising the AC domain (Ag43^UTI89^, residues 53–613 and Ag43^EDL933^, residues 53–613). The coding sequences for the α-domains of Ag43b, Ag43^UTI89^ and Ag43^EDL933^ were amplified from CFT073, UTI89 and EDL933, respectively (primers shown in Supplementary Table S3). These sequences were cloned into a modified version of a pMCSG7 expression vector encoding an N-terminal His_6_ tag with thioredoxin (TRX) followed by the tobacco-etch-virus (TEV) protease cleavage site^50^. The proteins were expressed in the host strain *E. coli* BL21(DE3)pLysS with the autoinduction method (25 °C for 20 h in the case of α^43b^ and 30 °C for 20 h for α^UTI89^ and α^EDL933^). For α^UTI89^ and α^EDL933^ only the longer constructs expressed at high yields and showed higher stability and thus were used for subsequent x-ray crystallography studies. The three proteins were purified by nickel affinity chromatography and TEV-cleaved. Uncleaved proteins were removed by a second round of nickel affinity chromatography. The α-domains of the proteins were purified to homogeneity by size exclusion chromatography (Superdex 75 GE Healthcare) equilibrated in 25 mM HEPES pH 7, 150 mM NaCl and purity was assessed by sodium dodecyl sulphate–polyacrylamide gel electrophoresis (SDS–PAGE).

### Crystallisation and diffraction data measurement

Purified α^43_UTI89^, α^43_EDL933^ and α^43b^ in 25 mM HEPES, 50 mM NaCl, pH 7 were concentrated to 15.6, 18.3 and 12 mg mL^−1^, respectively, and crystallised using the hanging-drop vapour diffusion method. Small block-like crystals were obtained for α^43_UTI89^ (0.10 × 0.20 × 0.10 mm) from a solution consisting of 20% 2-propanol, 100 mM trisodium citrate/citric acid pH 5.2, 20% PEG 4000. Large needle crystals were obtained for α^43_EDL933^ (0.7 × 0.05 × 0.05 mm) in 100 mM SPG (succinic acid, sodium dihydrogen phosphate and glycine) pH 7.4, 27% PEG 1500. Stacked plate crystals were obtained for α^43b^ (~0.5 × 0.25 × 0.05 mm) from a solution consisting of 100 mM Na cacodylate pH 6.4, 14% PEG 4000, 20% MPD.

Diffraction data were collected at the Microcrystallography MX2 beamline at the Australian Synchrotron using an ADSC Q315r CCD detector. 180° images were collected for crystals of the three proteins, at an oscillation angle of 1°, exposure time of 1 s. iMosflm^55^ and Aimless^56^ software was utilised to index, integrate and scale the collected diffraction data.

### Structure determination of Ag43 variants and refinement

The structure of α^43_UTI89^ and α^43b^ were solved with Phaser^57^ by molecular replacement using the structure of α^43a^ (PBD: 4KH3)^13^ as a model. Similarly, the structure of α^43_EDL933^ was solved via molecular replacement using α^43_UTI89^ as a reference. The corresponding protein models were manually built in Coot^58^, and refined with phenix.refine^59^ and translation/libration/screw (TLS) refinement^60^. Model quality was monitored by the *R*-free value, which represented 5% of the data. The models for all variants were evaluated by MolProbity^61^ and figures were generated using PyMOL^58,62^. Coordinates and structure-factor files for α^43_UTI89^, α^43b^ and α^43_EDL933^ have been deposited in the Protein Data Bank, with accession codes 7KO9, 7KOB, 7KOH, respectively. Data-processing and refinement statistics are summarised in Table 1.

### Analytical ultracentrifugation

Sedimentation velocity experiments were performed using a Beckman Optima XL-A analytical ultracentrifuge, 8-hole An-50 Ti rotor. Protein samples (380 µL) obtained after size exclusion chromatography of the recombinant Ag43 variants in 25 mM HEPES, 150 mM NaCl, pH 7.0 and reference buffer (400 µL) were loaded into double-sector quartz cells. Initial scans were performed at 3000 rpm to determine the optimal wavelength and radial positions. Absorbance readings were collected at 285 nm and 40,000 rpm at 20 °C. Solvent density, solvent viscosity and estimates of the partial specific volume of α^43b^ (0.7211 mL g^−1^), α^43_UTI89^ (0.7197 mL g^−1^), α^43a^ (0.7215 mL g^−1^) and α^43_EDL933^ (0.7207 mL g^−1^) at 20 °C were calculated with SEDNTERP^63^. Data were analysed using *c*(s) with SEDFIT^64^.

### Small angle X-ray scattering

SAXS data were collected on the SAXS/WAXS beamline at the Australian Synchrotron^65^. Approximately 60 μL of α^43a^ and α^43_UTI89^, at 1.2 mg mL^−1^, in 25 mM HEPES, 150 mM NaCl, pH 7.4 were loaded into a 1 mm quartz capillary. Samples were flowed during data collection to reduce radiation damage. Data reduction was carried out using the ScatterBrain software (v.2.71) and the data were corrected for solvent scattering and sample transmission, then radially averaged to produce *I*(*q*) as a function of *q*, where *q* = (4*π*sin *θ*)/*λ*, *θ* is half the scattering angle, and *λ* is the X-ray wavelength. For α^43a^ (SASBDB ID: SASDKQ3) and α^43_UTI89^ (SASBDB ID: SASDKP3), data were fit as a linear combination of model curves to quantify the nature and proportions of different oligomeric states in solution^66^. Model scattering curves were calculated using CRYSOL (v.2.8.3)^67^, using monomer and dimer structures taken from PDB: 4KH3 for α^43a^ and PDB: 7KO9 for α^43_UTI89^.

### Reporting summary

Further information on research design is available in the Nature Research Reporting Summary linked to this article.

## Supplementary information

Reporting Summary Checklist

Supplemental Material

## Data Availability

The crystallography, atomic coordinates, and structure factors reported in this paper have been deposited in the Protein Data Bank, www.pdb.org (PDB ID codes 7KO9, 7KOB and 7KOH). The Small Angle X-ray Scattering data reported in this paper have been deposited in the Small Angle X-ray Scattering Biological Data Bank, www.sasbdb.org (SASBDB ID codes: SASDKP3 and SASDKQ3).
