# Supplementary material for: Variation of Antigen 43 self-association modulates bacterial compacting within aggregates and biofilms
Source: NPJ Biofilms Microbiomes. 2022 Apr 8;8:20. doi: 10.1038/s41522-022-00284-1 (PMC8993888; doi:10.1038/s41522-022-00284-1)
Supplement: Supplementary file 1 — Reporting Summary Checklist [file 41522_2022_284_MOESM1_ESM.pdf]

nature portfolio

Corresponding author(s): Begona Heras, Jason Paxman, Mark Schembri

Last updated by author(s): 2022/02/09

Reporting Summary

Nature Portfolio wishes to improve the reproducibility of the work that we publish. This form provides structure for consistency and transparency in reporting. For further information on Nature Portfolio policies, see our Editorial Policies and the Editorial Policy Checklist.

Statistics

For all statistical analyses, confirm that the following items are present in the figure legend, table legend, main text, or Methods section.

n/a	Confirmed	
☐	☒	The exact sample size (*n*) for each experimental group/condition, given as a discrete number and unit of measurement
☐	☒	A statement on whether measurements were taken from distinct samples or whether the same sample was measured repeatedly
☐	☒	The statistical test(s) used AND whether they are one- or two-sided<br>*Only common tests should be described solely by name; describe more complex techniques in the Methods section.*
☐	☒	A description of all covariates tested
☒	☐	A description of any assumptions or corrections, such as tests of normality and adjustment for multiple comparisons
☐	☒	A full description of the statistical parameters including central tendency (e.g. means) or other basic estimates (e.g. regression coefficient) AND variation (e.g. standard deviation) or associated estimates of uncertainty (e.g. confidence intervals)
☐	☒	For null hypothesis testing, the test statistic (e.g. F, t, r) with confidence intervals, effect sizes, degrees of freedom and P value noted<br>*Give P values as exact values whenever suitable.*
☒	☐	For Bayesian analysis, information on the choice of priors and Markov chain Monte Carlo settings
☒	☐	For hierarchical and complex designs, identification of the appropriate level for tests and full reporting of outcomes
☒	☐	Estimates of effect sizes (e.g. Cohen's *d*, Pearson's *r*), indicating how they were calculated

Our web collection on statistics for biologists contains articles on many of the points above.

Software and code

Policy information about availability of computer code

Data collection	X-ray diffraction data were collected using the Blue Ice software. (McPhillips et al. J. Synchrotron Rad. (2002). 9, 401-406)<br>SAXS data were collected on the SAXS/WAXS beamline at the Australian Synchrotron

Data analysis

Protein structural alignments
- DALI server (http://ekhidna.biocenter.helsinki.fi/dali_server/start)

Protein structure determination by X-ray crystallography
- Data were integrated, scaled and merged using iMosflm (Battye TGG, et al. Acta Crystallographica Section D: Biological Crystallography. 2011;67:271-81) and Aimless (Evans PR, Murshudov GN. Acta Crystallographica Section D: Biological Crystallography. 2013;69:1204-14)
.
- Structures solved using Phaser (McCoy AJ, et al J Appl Crystallogr. 2007;40(Pt 4):658-74).

- Model building COOT (Emsley, P. & Cowtan, K. Acta Crystallogr D Biol Crystallogr 60, 2126-32 2004).

- Protein structure refinement Phenix.refine (Adams, P.D. et al. Acta Crystallogr D Biol Crystallogr 58, 1948-54 2002).

- Protein structure validation MolProbity (Davis, I.W. et al. Nucleic Acids Res 35, W375-83 2007).

- Protein structure figures were created with PyMOL (DeLano, W.L. The PyMOL Molecular Graphics System, http://www.pymol.org/ . DeLano Scientific, San Carlos, CA, USA. 2002

- SAXS Model scattering curves calculated using CRYSOL (v.2.8.3) Svergun D, Barberato C, Koch MHJ. Journal of Applied Crystallography. 1995;28(6):768-73.

For manuscripts utilizing custom algorithms or software that are central to the research but not yet described in published literature, software must be made available to editors and reviewers. We strongly encourage code deposition in a community repository (e.g. GitHub). See the Nature Portfolio guidelines for submitting code & software for further information.

Data

Policy information about availability of data

All manuscripts must include a data availability statement. This statement should provide the following information, where applicable:
- Accession codes, unique identifiers, or web links for publicly available datasets
- A description of any restrictions on data availability
- For clinical datasets or third party data, please ensure that the statement adheres to our policy

The crystallography, atomic coordinates, and structure factors reported in this paper have been deposited in the Protein Data Bank, www.pdb.org (PDB ID codes 7KO9, 7KOB and 7KOH).
The Small Angle X-ray Scattering data reported in this paper have been deposited in the Small Angle X-ray Scattering Biological Data Bank, www.sasbdb.org (SASBDB ID codes: SASDKP3 and SASDKQ3).

Field-specific reporting

Please select the one below that is the best fit for your research. If you are not sure, read the appropriate sections before making your selection.

☒ Life sciences ☐ Behavioural & social sciences ☐ Ecological, evolutionary & environmental sciences

For a reference copy of the document with all sections, see nature.com/documents/nr-reporting-summary-flat.pdf

Life sciences study design

All studies must disclose on these points even when the disclosure is negative.

Sample size	Sample sizes were not predetermined based on statistical methods, but were chosen according to the standards in the field - at least 3 independent biological replicates for each condition. Where appropriate, this generated sufficient data for statistical analysis.
Data exclusions	No data was excluded from this manuscript
Replication	Reported results were consistently replicated across multiple experiments with all replicates generating similar results.
Randomization	No randomization was necessary as experiments were performed with appropriate controls. Randomization is not generally used in this field.
Blinding	Investigators were not blinded. Blinding during analysis was not necessary because the results are quantitative and did not require subjective judgment or interpretation. Blinding is not typically used in the field.

Reporting for specific materials, systems and methods

We require information from authors about some types of materials, experimental systems and methods used in many studies. Here, indicate whether each material, system or method listed is relevant to your study. If you are not sure if a list item applies to your research, read the appropriate section before selecting a response.

Materials & experimental systems

n/a	Involved in the study	
☐	☒	Antibodies
☒	☐	Eukaryotic cell lines
☒	☐	Palaeontology and archaeology
☒	☐	Animals and other organisms
☒	☐	Human research participants
☒	☐	Clinical data
☒	☐	Dual use research of concern

Methods

n/a	Involved in the study	
☒	☐	ChIP-seq
☐	☒	Flow cytometry
☒	☐	MRI-based neuroimaging

Antibodies

Antibodies used: Rabbit polyclonal serum against α43a, α43-EDL933 and α43-UTI89 was generated at the WEHI antibody facility, Melbourne, Australia. Anti-rabbit-AP secondary antibody was purchased from Sigma-Aldrich (catalogue number A3687). Anti-rabbit IgG/ HRP antibody was purchased from Promega (W401B).

Validation: α43a, α43-EDL933 and α43-UTI89 antisera were validated by Western Blotting using purified recombinant Ag43 passenger-domain protein. Detection with anti-rabbit-AP secondary antibody validated its species-specific source.

Flow Cytometry

Plots

Confirm that:

☒ The axis labels state the marker and fluorochrome used (e.g. CD4-FITC).

☒ The axis scales are clearly visible. Include numbers along axes only for bottom left plot of group (a 'group' is an analysis of identical markers).

☒ All plots are contour plots with outliers or pseudocolor plots.

☐ A numerical value for number of cells or percentage (with statistics) is provided.

Methodology

Sample preparation: E. coli strains harboring plasmids encoding Ag43a, Ag43b or the control plasmid pBAD/Myc-HisA were used. Strains were cultured overnight in LB broth, washed with PBS and resuspended in and equal volume of PBS. All sample dilutions were performed in PBS.

Instrument: BD Accuri C6 flow cytometer (BD Bioscience, San Diego, CA, USA)

Software: FlowJo 10.X.7 (Tree Star)

Cell population abundance: Not relevant. Each bacterial strain was examined individually.

Gating strategy: No gating strategy was used. The bacterial population was examined using forward scatter versus side scatter. Readings were collected in logarithmic mode comprising at least 5000 events per sample. Bacterial cell aggregation was reflected by increased forward scatter.

☐ Tick this box to confirm that a figure exemplifying the gating strategy is provided in the Supplementary Information.
