# Supplementary material for: Variation of Antigen 43 self-association modulates bacterial compacting within aggregates and biofilms
Source: NPJ Biofilms Microbiomes. 2022 Apr 8;8:20. doi: 10.1038/s41522-022-00284-1 (PMC8993888; doi:10.1038/s41522-022-00284-1)
Supplement: Supplementary file 2 — Supplemental Material [file 41522_2022_284_MOESM2_ESM.pdf]

Supplementary Information

Variation of Antigen 43 self-association modulates bacterial compacting within aggregates and biofilms

Julieanne L Vo[1,6], Gabriela C Martínez Ortiz[1,6], Makrina Totsika[2], Alvin Lo[3], Steven J Hancock[3], Andrew E. Whitten[4], Lilian Hor[1], Kate M Peters[3], Valentin Ageorges[5], Nelly Caccia[5], Mickaël Desvaux[5], Mark A Schembri[3,✉], Jason J Paxman[1,✉] and Begoña Heras[1,✉]

[1]Department of Biochemistry and Genetics, La Trobe Institute for Molecular Science, La Trobe University, Melbourne VIC 3086, Australia; [2]Centre for Immunology and Infection Control, School of Biomedical Sciences, Queensland University of Technology, Herston, QLD 4006, Australia; [3]Australian Infectious Diseases Research Centre, School of Chemistry and Molecular Biosciences, The University of Queensland, Brisbane QLD 4072, Australia; [4]Australian Centre for Neutron Scattering, Australian Nuclear Science and Technology Organisation, Lucas Heights, NSW 2234, Australia; [5]Université Clermont Auvergne, INRAE, UMR454 MEDiS, 63000, Clermont-Ferrand, France. [6]These authors contributed equally: Julieanne L Vo, Gabriela C Martínez Ortiz. ✉email: m.schembri@uq.edu.au, ✉email: j.paxman@latrobe.edu.au, ✉email:b.heras@latrobe.edu.au.

Figures Supplementary 1 to 8

Tables Supplementary 1 to 4

Supplementary References

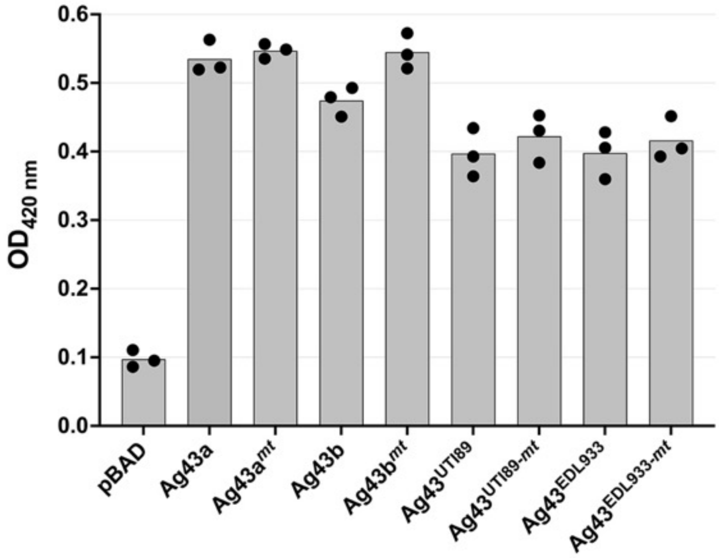

Supplementary Figure 1. Whole-cell ELISA of *E. coli* MS528 cells expressing native and mutant Ag43a, Ag43b, $Ag43^{UTI89}$ and $Ag43^{EDL933}$ proteins. ELISA plate wells were coated with cell suspensions and after blocking, they were incubated with rabbit polyclonal serum α^{43a} antibody followed by incubation with an anti-rabbit secondary antibody and development with pNPP substrate. Absorbance was measured at 420 nm. Similar levels of surface expression were detected for all native and mutant proteins. Three biological replicates of each strain were measured with four technical replicates. Data are shown as the mean ± standard deviation of three replicates.

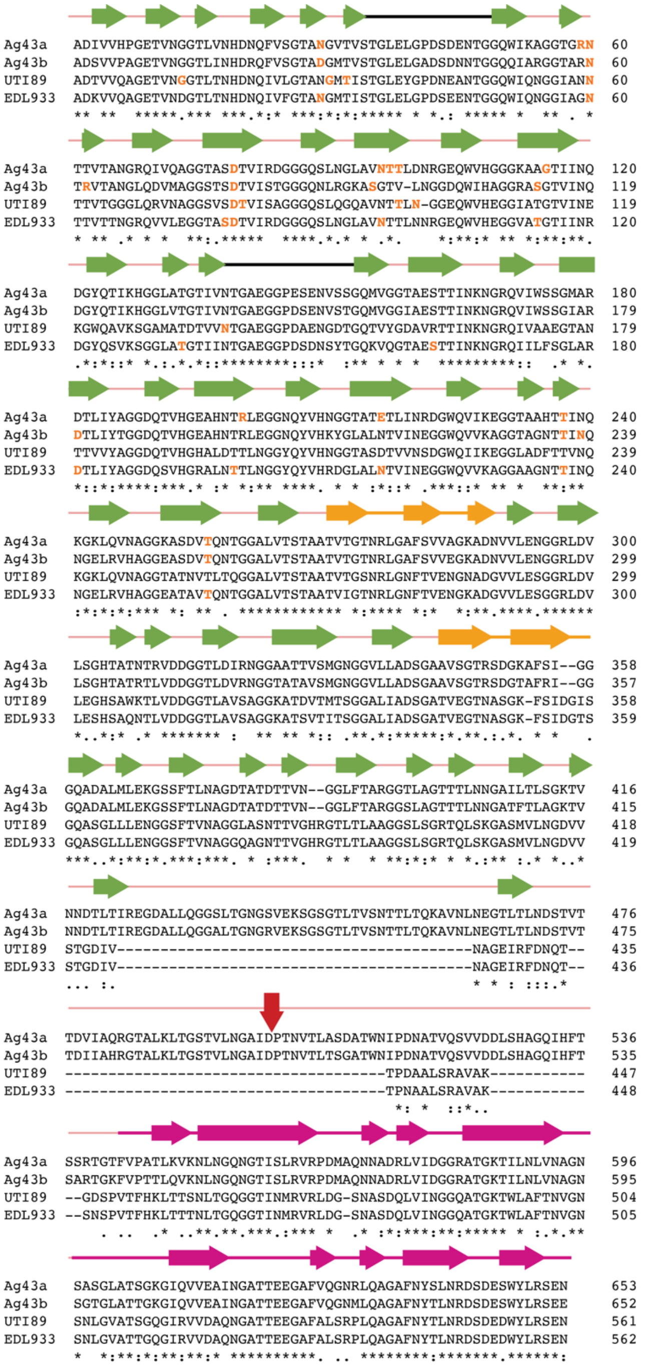

Supplementary Figure 2. Multiple sequence alignment of Ag43a, Ag43b, Ag43^{UTI89} and Ag43^{EDL933} passenger domains. The mature form of the passenger domain of Ag43a, Ag43b, Ag43^{UTI89} and Ag43^{EDL933}, lacking the signal sequence and encompassing the α-domain (α^{43a}, α^{43b}, α^{43_UTI89} and $\alpha^{43\_ EDL933}$) and auto-chaperone domain (AC^{43a}, AC^{43b} AC^{43_UTI89} and AC^{43_EDL933}) were aligned with Clustal Omega. Secondary structural elements are based on the structure of α^{43_EDL933}, following the colour coding of Fig 2A: β-strands illustrated in green and loops depicted in salmon. The two protruding loops are shown in black, the β-hairpins appear in cyan and the region coloured in hot pink corresponds to the AC domain. Interface residues are indicated in bold orange font. The red arrow shows the cleavage site between α^{43a} and AC^{43a} and α^{43b} and AC^{43b}.

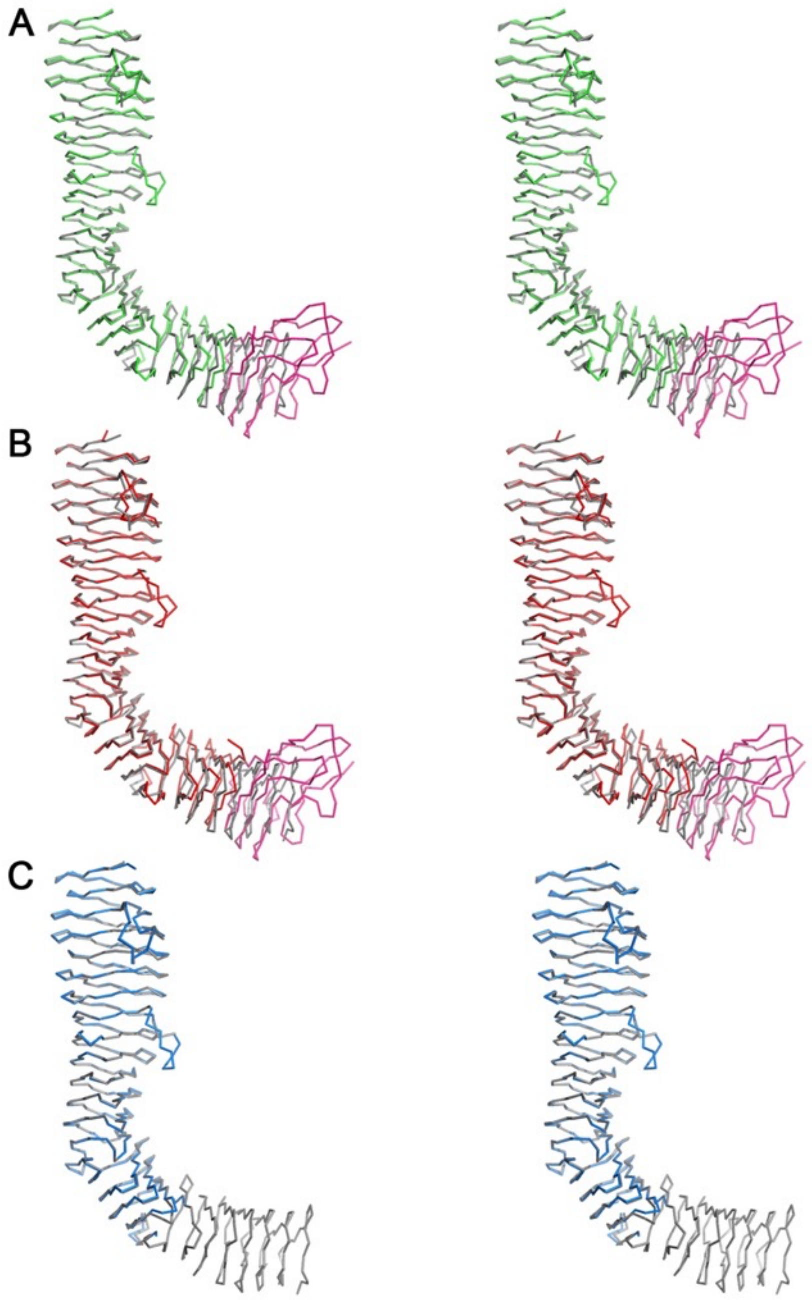

Supplementary Figure 3. Structural alignment of Ag43a, Ag43b, Ag43^{UTI89} and Ag43^{EDL933} passenger domains. Stereo view of the α-carbon trace superposition of α^{43a} (grey) with, (A) α^{43_EDL933} (green), (B) α^{43_UTI89} (red) and (C) α^{43b} (blue). Auto chaperone domain in α^{43_EDL933} and α^{43_UTI89} shown in hot pink.

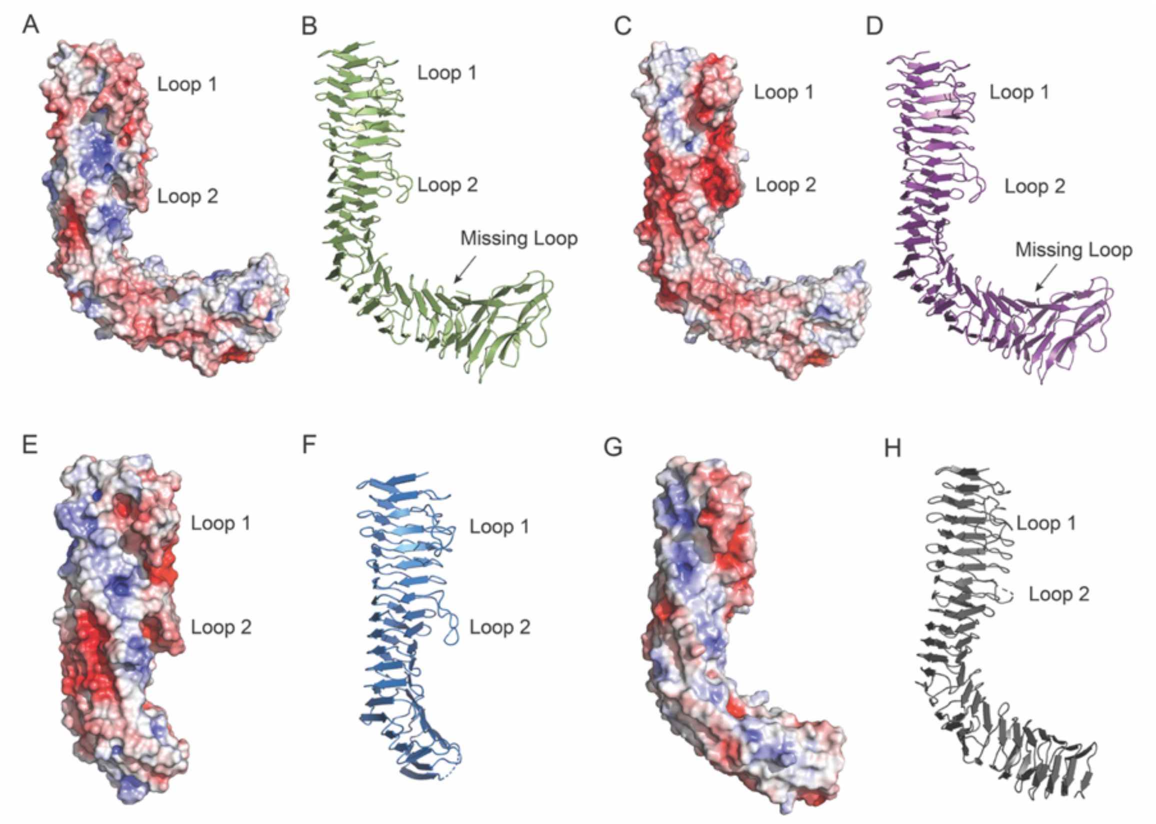

Supplementary Figure 4. Electrostatic surface representation of α^{43_EDL933}, α^{43_UTI89}, α^{43b} and α^{43a} (A, C, E, G) Electrostatic surface representation of α^{43_EDL933}, α^{43_UTI89} and α^{43b} respectively, along with the previously published α^{43a} (PDB: 4KH3) for comparison. For each protein, positive electrostatic potentials are shown in blue, while negative electrostatic potentials appear in red (saturation at 5 kT/e). The two loops (Loop 1 and Loop 2) that protrude from the β-helices reveal acidic patches in these negatively charged loops. (B, D, F, H) Cartoon representation of α^{43_EDL933}, α^{43_UTI89}, α^{43b} and α^{43a} respectively showing the orientation of all proteins in panels A, C, E and G.

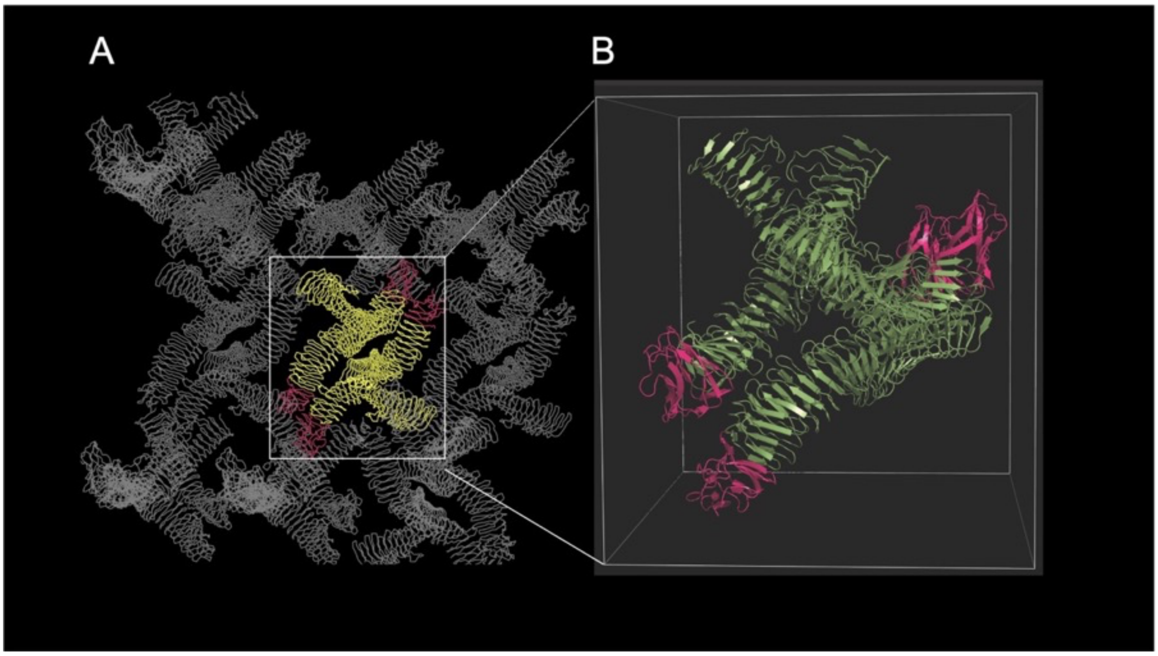

Supplementary Figure 5. (A) Crystal lattice of α^{43_EDL933} with non-biologically relevant crystal contacts observed between the molecules. (B) A zoomed view of α^{43_EDL933}, showing the four molecules present in its asymmetric unit. The α-domains are depicted in green and the AC domains are displayed in hot pink.

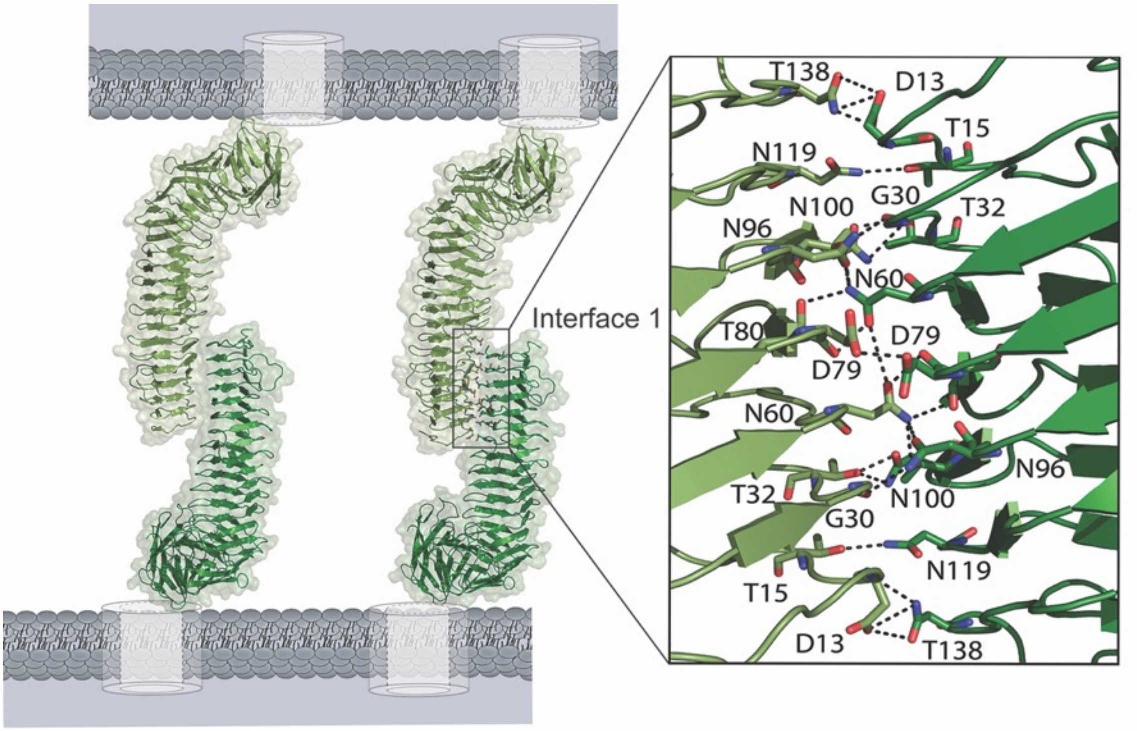

Supplementary Figure 6. α^{43_EDL933}- α^{43_EDL933} dimeric single-interface. Self-association of α^{43_EDL933} molecules on the bacterial cell surface, showing a single interface predicted using α^{43_UTI89} dimer as a model. A close-up view of the interaction interface is shown; the interface consists of 24 hydrogen bonds [D13-N138 (three hydrogen bonds), T15-N119, G30-T98, T32-N100 (two hydrogen bonds), N60-N96, N60-T98, N60-T80, D79-N60, N60-N60, D79-D79, D79-N60, T80-N60, T98-N60, T98-G30, N96-N60 N100-T32 (two hydrogen bonds), N119-T15, N138-D13 (three hydrogen bonds)]. This dimer forms through self-interaction via the F3 face of α^{43_EDL933}.

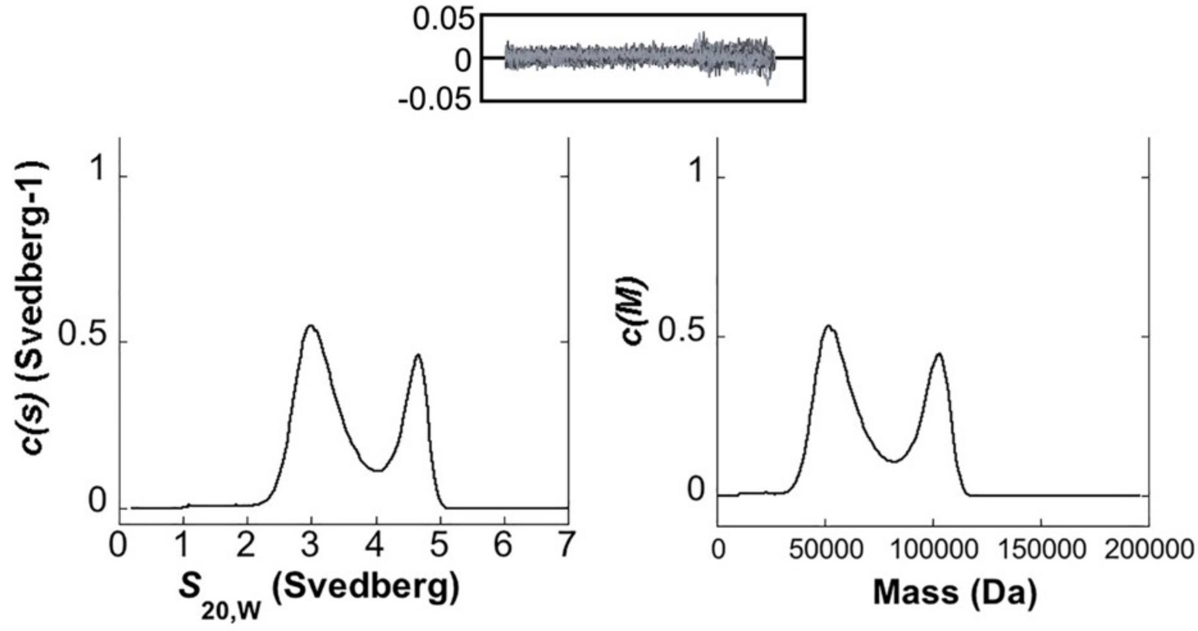

Supplementary Figure 7. Ag43b can form dimers in solution. Analytical ultracentrifugation (AUC) sedimentation velocity analysis of α^{43b} with *c(s)* plotted as a function of $s_{20,w}$ (Svedberg) and *c(M)* plotted as a function of mass. In this experiment Ag43b was prepared at higher initial concentrations (> 30 mg/ml) when compared to the samples in Figure 5. As shown α^{43b} has the capacity to form both monomers 2.8 S (50 kDa) and dimers 4.6 S (100 kDa). Residuals resulting from the *c*(*s*) distribution fit is shown above.

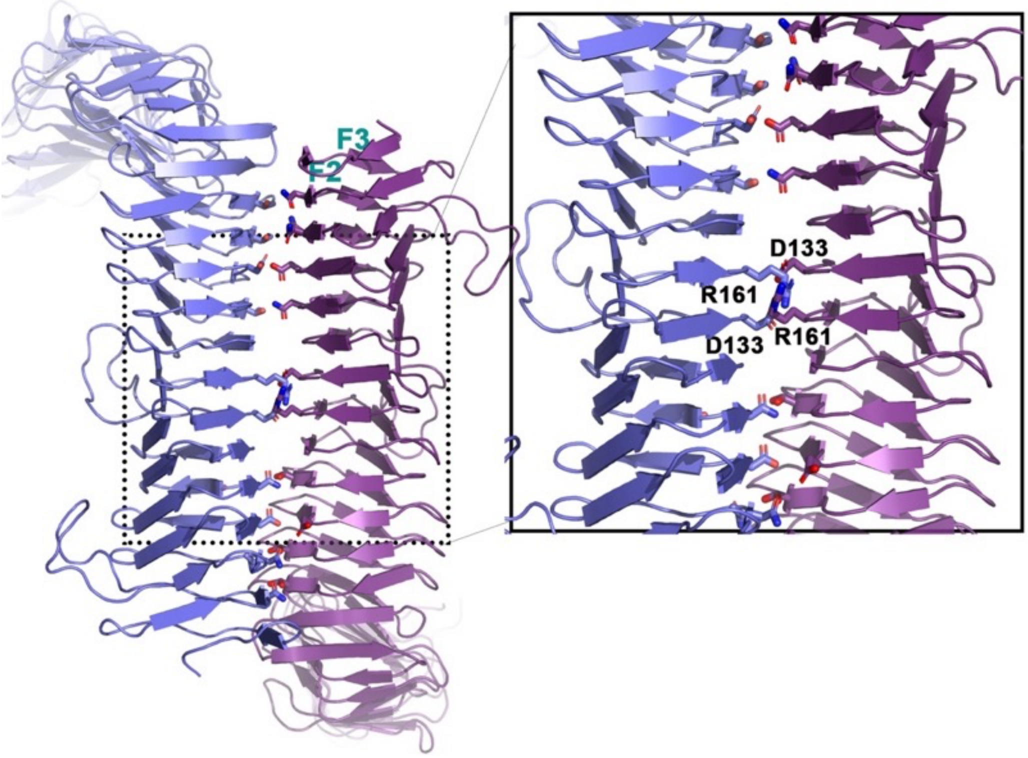

Supplementary Figure 8. Model of α$^{43_UTI-89}$- α$^{43_UTI-89}$ double-interface interaction.
Double interface self-association of α^{43_UTI89} modelled on α^{43a}- α^{43a} dimer showing the R161 residues in the F2-F3 loops. The long sidechains of D133 and R161 in the lower part of the β-helix would result in steric clashes between interacting proteins preventing a head-to-tail association via a double interface.

Supplementary Table 1. Comparison of the structures of Ag43 passenger (α) domains and Ag43 autochaperone (AC) domains with reference autotransporter AC domains.

	α^{43a} (492)[d]	α^{43_UTI89} (540)[d]	α^{43_EDL933} (545)[d]	α^{43b} (361)[d]	$\alpha^{43\_EDL933\text{-}AC}$ (109)[d]	$\alpha^{43\_UTI89\text{-}AC}$ (109)[d]	$\alpha^{EspP\text{-}AC}$ (131)[d]	$\alpha^{Hap\text{-}AC}$ (124)[d]	$\alpha^{Hbp\text{-}AC}$ (97)[d]	$\alpha^{IcsA\text{-}AC}$ (135)[d]	$\alpha^{P69\text{-}AC}$ (96)[d]
α^{43_EDL933} (545)[d]	2.09[a] (413)[b] (64.6%)[c]	2.07[a] (493)[b] (74.6%)[c]	0[a] (545)[b] (100%)[c]	0.58[a] (354)[b] (71.2%)[c]							
α^{43_UTI89} (540)[d]	1.62[a] (455)[b] (57.6%)[c]	0[a] (540)[b] (100%)[c]	2.07[a] (493)[b] (74.6%)[c]	2.54[a] (306)[b] (50.3%)[c]							
α^{43b} (361)[d]	0.52[a] (350)[b] (80.9%)[c]	2.54[a] (306)[b] (50.3%)[c]	0.58[a] (354)[b] (71.2%)[c]	0[a] (361)[b] (100%)[c]							
$\alpha^{43\_EDL933\text{-}AC}$ (109)[d]					0[a] (109)[b] (100%)[c]	0.38[a] (109)[b] (98.2%)[c]	1.50[a] (82)[b] (26.8%)[c]	2.87[a] (77)[b] (26%)	4.37[a] (21)[b] (19%)[c]	2.53[a] (92)[b] (27.2%)[c]	2.38[a] (61)[b] (18%)[c]
$\alpha^{43\_UTI89\text{-}AC}$ (109)[d]					0.38[a] (109)[b] (98.2%)[c]	0[a] (109)[b] (100%)	3.15[a] (70)[b] (10%)[c]	2.82[a] (76)[b] (26.3%)[c]	4.19[a] (21)[b] (19%)[c]	2.57[a] (92)[b] (27.2%)[c]	2.77[a] (69)[b] (16%)[c]

[a]RMSD (Root Mean Square Deviation) values (Å) calculated using Secondary Structure Matching (SSM) superimpose tool in Coot (2)
[b] number of aligned Cα atoms
[c] sequence identity
[d] total number of residues
Aligned structures: Ag43a (PDB: 4KH3), $Ag43^{UTI89}$ (PDB: 7KO9), $Ag43^{EDL933}$ (PDB: 7KOH), Ag43b (PDB: 7KOB), $Ag43^{EDL933}$ (PDB: 7KOH; AC: V_{453}-E_{561}), $Ag43^{UTI89}$ (PDB: 7KO9; AC: V_{452}-E_{560}), EspP (PDB: 3SZE; AC: D_{869}-A_{999}), Hap (PDB: 3SYJ; AC: D_{830}-P_{976}), Hbp (PDB: 1WXR; AC: V_{946}-N_{1048}), P69 (PDB: 1DAB; AC: L_{444}-P_{539}) and IcsA (PDB: 3ML3; AC: D_{606}-D_{740})

Supplementary Table 2 SAXS data collection details.

	α^{43a}	α^{43_UTI89}
SASBDB ID	SASDKQ3	SASDKP3
Data Collection Parameters		
Instrument	SAXS-WAXS, Australian Synchrotron	SAXS-WAXS, Australian Synchrotron
Beam geometry (μm)	80 × 200	80 × 200
Wavelength (Å)	1.0332	1.0332
Flux (photons/s)	3.1×10^{12}	3.1×10^{12}
Sample to detector distance (m)	2.680	1.428
q-range (Å^{-1})	0.00 –0.30	0.011–0.60
Temperature (K)	285	285
Absolute intensity calibration	Water	Water
Exposure time (s)	14 (14 × 1 s exposures)	35 (35 × 1 s exposures)
Configuration	Single measurement from 96-well plate	Single measurement from 96-well plate
Protein concentration (mg ml^{-1})	1.2	1.2

Supplementary Table 3. Primers, plasmids and strains used in this study.

	**Details**	**Reference**
**Primer**		
UTI89_c1139a_Lic_Fw	TACTTCCAATCCAATGCGGCTGACACGGTTGTACAG	This study
UTI89_c1139a_Lic_Rv	TTATCCACTTCCAATGTTCACTGCGCAGATACCA	This study
UTI89_c1139a_Lic_Rv (short)	TTATCCACTTCCAATGGCGAATCTCTCCGGCGTT	This study
EDL933_z1211a_Lic_Fw	TACTTCCAATCCAATGCGGCTGACAAGGTTGTACAG	This study
EDL933_z1211a_Lic_Rv	TTATCCACTTCCAATGTTCACTGCGCAGATACCA	This study
EDL933_z1211a_Lic_Rv (short)	TTATCCACTTCCAATGGCGAATCTCCCCTGCGTT	This study
Ag43a and Ag43b Fw	GGGTAAAGCTGATAATGTCG	This study
Ag43a and Ag43b Rv	GTTGCTGACAGTGAGTGTGC	This study
FL UTI89 FW	CGCGCTCGAGATAATAAGGAAAAGCTGATGAAAC	This study
FL UTI89 Rv	GGCCCAAGCTTCTGTCAGAAAGTCATATTCAGCG	This study
FL EDL933 Fw	CGCGCTCGAGATAATAAGGAAAAGCTGATGAAAC	This study
FL EDL933 Rv	GGCCCAAGCTTCTGTCAGAAAGTCATATTCAGCG	This study
**Plasmids**		
pBAD/Myc-His A	Plasmid used for expression of full-length proteins	(1)
pMCSG7	Plasmid used for expression of Ag43 functional α-domains	(2)
**Strains**		
MS528	*E. coli* MG1655 *fim agn43* null strain	(3)
OS56	*E. coli* MG1655 *agn43* null strain GfP^{+}	(4)

Supplementary Table 4. Residues mutated for mutant design of proteins.

**Protein**	**Residues mutated to Glycine**
Ag43$^{UTI89\text{-}mt}$	T32, N60, D79, T80, T98, N100, N137
Ag43b^{mt}	D29, N60, R62, D79, S95, S113
Ag43$^{EDL933\text{-}mt\text{-}single}$	T15, T32, N60, D79, T98, N100, N119, N137
Ag43$^{EDL933\text{-}mt\text{-}double}$	T199, T256

Supplementary References

1. Guzman LM, Belin D, Carson MJ, Beckwith J. Tight regulation, modulation, and high-level expression by vectors containing the arabinose PBAD promoter. J Bacteriol. 1995;177(14):4121-30.
2. Paxman JJ, Lo AW, Sullivan MJ, Panjikar S, Kuiper M, Whitten AE, et al. Unique structural features of a bacterial autotransporter adhesin suggest mechanisms for interaction with host macromolecules. Nature Communications. 2019;10(1):1967.
3. Klemm P, Hjerrild L, Gjermansen M, Schembri MA. Structure-function analysis of the self-recognizing Antigen 43 autotransporter protein from Escherichia coli. Molecular Microbiology. 2004;51(1):283-96.
4. Ulett GC, Valle J, Beloin C, Sherlock O, Ghigo JM, Schembri MA. Functional analysis of antigen 43 in uropathogenic Escherichia coli reveals a role in long-term persistence in the urinary tract. Infection and immunity. 2007;75(7):3233-44.
